# SU(2) hadrons on a quantum computer via a variational approach

**DOI:** 10.1038/s41467-021-26825-4

**Published:** 2021-11-11

**Authors:** Yasar Y. Atas, Jinglei Zhang, Randy Lewis, Amin Jahanpour, Jan F. Haase, Christine A. Muschik

**Affiliations:** 1 Institute for Quantum Computing, University of Waterloo, Waterloo, ON Canada N2L 3G1; 2 Department of Physics & Astronomy, University of Waterloo, Waterloo, ON Canada N2L 3G1; 3 Department of Physics and Astronomy, York University, Toronto, ON Canada M3J 1P3; 4 Institut für Theoretische Physik und IQST, Universität Ulm, Albert-Einstein-Allee 11, D-89069 Ulm, Germany; 5 Perimeter Institute for Theoretical Physics, Waterloo, ON Canada N2L 2Y5

**Keywords:** Theoretical nuclear physics, Quantum simulation

## Abstract

Quantum computers have the potential to create important new opportunities for ongoing essential research on gauge theories. They can provide simulations that are unattainable on classical computers such as sign-problem afflicted models or time evolutions. In this work, we variationally prepare the low-lying eigenstates of a non-Abelian gauge theory with dynamically coupled matter on a quantum computer. This enables the observation of hadrons and the calculation of their associated masses. The SU(2) gauge group considered here represents an important first step towards ultimately studying quantum chromodynamics, the theory that describes the properties of protons, neutrons and other hadrons. Our calculations on an IBM superconducting platform utilize a variational quantum eigensolver to study both meson and baryon states, hadrons which have never been seen in a non-Abelian simulation on a quantum computer. We develop a hybrid resource-efficient approach by combining classical and quantum computing, that not only allows the study of an SU(2) gauge theory with dynamical matter fields on present-day quantum hardware, but further lays out the premises for future quantum simulations that will address currently unanswered questions in particle and nuclear physics.

## Abstract (web summary)

Quantum simulations of lattice gauge theories are in principle scalable, but their extension to dynamically coupled matter has proven difficult. In this work, the authors use a variational quantum eigensolver to simulate a non-Abelian LGT including the effects of both gauge fields and dynamical fermions.

## Introduction

Quantum computing technologies are developing quickly in recent years with applications in a broad range of scientific areas from chemistry to fundamental interactions of Nature. Prime candidates for the application of such quantum simulations are gauge theories, which play a major role in many branches of physics and comprise the entire Standard Model of particle physics. Within this area, quantum computation of non-Abelian gauge theories is an outstanding challenge.

The most prominent example, quantum chromodynamics (QCD), is a non-Abelian gauge theory that explains the strong interactions between quarks and gluons and ultimately underlies nuclear physics. There are also suggestions of non-Abelian forces beyond the Standard Model (BSM) that are completely separate from QCD and might, for example, underlie the Higgs sector of the Standard Model^1^ or provide a strongly interacting theory for dark matter^2^.

Lattice gauge theory (LGT)^3^ is a mature and successful discretisation strategy for computational methods that have developed into an extremely successful field of science. The formulation of the theory on a spacetime lattice provides a non-perturbative regularization of the theory with the lattice spacing playing the role of an inverse UV cutoff. Modern LGT calculations have provided precise quantitative results and important insights for QCD^4^, nuclear physics^5^, and non-Abelian BSM theories^6^, and they will continue to do so for the foreseeable future. Quantum computers offer a possibility to extend the reach of LGT into regimes that are presently unattainable^7^. Fundamental issues like the sign problem^8,9^ prevent classical methods for LGTs from studying many properties of interest such as real-time particle dynamics and highly entangled matter, so quantum simulations will play an essential role in improving our understanding of Nature.

Quantum simulations of LGTs are a growing research area^10^, addressing both real-time dynamics and equilibrium problems. Our work contributes to the latter. Equilibrium problems include important sign-problem afflicted settings such as models with high matter density (with finite chemical potentials) and topological theories. While current proof-of-concept demonstrations of equilibrium problems still address sign-problem-free settings, they form the foundation for extensions to more complicated models. This foundation is currently built by simulating low-dimensional benchmarking models. Even though the ultimate goal is the simulation of three-dimensional (3D) theories, so far all quantum simulation experiments realize 1D models. Moreover, while different experimental realisations of 1D Abelian LGTs have been successful^11–16^, non-Abelian theories are fundamentally different. Efforts to confront this challenge are underway^17–31^, and a first important step has been made by experimentally realising pure gauge non-Abelian theories^32,33^. In this work, we present the first quantum computer calculation for a non-Abelian gauge theory with the dynamically coupled matter.

We consider as gauge group SU(2), which is the smallest non-Abelian Lie group and is thus a key step towards studying full QCD. In contrast to an Abelian theory, it is possible to build gauge singlet states from valence fermions, without any valence antifermions; the lowest energy state that exhibits this distinctly non-Abelian feature is called a baryon and it has no counterpart in an Abelian theory. The non-Abelian theory also contains a meson, which is built from one valence fermion and one valence antifermion and is thus the counterpart to a neutral state in an Abelian theory.

While the considered non-Abelian model can in principle be realized by adapting the very successfully explored purely quantum simulations^11,15,16,34,35^, its complexity is currently out of reach for implementing such strategies on present-day devices. We, therefore, use a hybrid quantum-classical approach and employ a so-called variational quantum eigensolver (VQE). Within the VQE protocols, the task of preparing the baryon and meson state is cast into the form of an optimisation problem which is solved by a classical algorithm with cost function evaluations made on a quantum co-processor.

Running deep quantum circuits on present-day devices is a formidable challenge in the current era of noisy intermediate-scale quantum (NISQ)-devices^36^, which pose severe restrictions in the number of qubits used and the number of gates applied. Given these restrictions, we use a number of measures to make the calculations possible: (i) We integrate out the gauge field degrees of freedom to reduce the experimental resources needed. (ii) We design efficient circuits that generate an ansatz state containing only components relevant for the chosen parameter regimes and (iii) we reduce the depth of the experimental circuit by relegating part of the computation to classical preprocessing that can be performed efficiently. Note that the techniques developed in points (ii) and (iii) are general and not exclusive to the studied non-Abelian theory or LGT calculations.

In this work we study an SU(2) gauge theory with dynamical matter fields on the IBM Quantum Experience^37,38^, and we experimentally study physics beyond the Abelian features demonstrated so far. More specifically, we perform a quantum computation experiment to variationally access the lowest hadron energies of the model, namely the non-Abelian baryon and the meson state. This allows us to calculate their masses on the quantum computer. In particular, we perform calculations for different lattice sizes to show how a known physical symmetry emerges: the baryon and meson masses are equal in the physical limit where lattice artifacts vanish.

## Results

### SU(2) gauge theory

The quantum field theory for SU(2) gauge fields interacting with fundamental fermions is well known^39^. At each point in spacetime a matter field operator can annihilate a fermion of one of two possible colors (here named red and green), or it can create the corresponding antiparticle. The gauge fields (or “gluons”) at each point mediate the interactions between color charges. The quantum field theory in the continuum is described in more detail in Supplementary Note 1.

Because the non-Abelian nature of SU(2) leads to strong interactions and the confinement of color charge, these fermions and gluons are confined within color-singlet hadrons that cannot be studied perturbatively. In order to access the non-perturbative regime, both classical and quantum simulations require formulating the gauge theory on a lattice. Lattice calculations on classical computers are successful in Euclidean spacetime with a least-action approach, but quantum computers can address new regimes of the theory by working directly in Minkowski spacetime with a Hamiltonian approach.

**Fig. 1:**
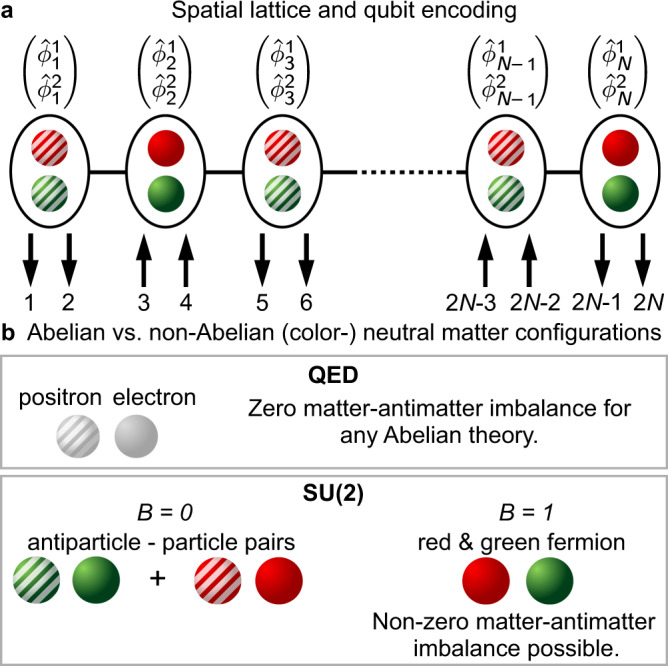
Gauge theory on a lattice. To study the SU(2) theory in one dimension, we employ the spatial lattice in (**a**), where each site consists of either matter or antimatter particles of the two possible colors. In the equivalent qubit formulation, each particle is represented by a qubit on a one-dimensional chain, which hence contains a number of qubits that equals twice the number of staggered sites. For a full discussion of the qubit representation see Supplementary Fig. 1. **b** Illustrates a comparison between the different gauge invariant states allowed in the neutral charge sector of Abelian QED and SU(2). While in the Abelian case neutral states require an equal number of matter (full spheres) and antimatter (striped spheres) particles, in the non-Abelian case, color-neutral states with a non-zero matter-antimatter imbalance are possible.

We follow the staggered fermion formulation of Kogut and Susskind, where fermions and antifermions occupy separate lattice sites, arranged in an alternating pattern along the lattice (Fig. 1a). The lattice Hamiltonian^40,41^ in natural units (*ℏ* = *c* = 1) is

1 $${\hat{H}}_{l}=\, \frac{1}{2{a}_{l}}\mathop{\sum }\limits_{n=1}^{N-1}\left({\hat{\phi }}_{n}^{{{{\dagger}}} }{\hat{U}}_{n}{\hat{\phi }}_{n+1}+{{{{{{{\rm{H}}}}}}}}.{{{{{{{\rm{C}}}}}}}}.\right)\\ +\ m\mathop{\sum }\limits_{n=1}^{N}{(-1)}^{n}{\hat{\phi }}_{n}^{{{{\dagger}}} }{\hat{\phi }}_{n}+\frac{{a}_{l}{g}^{2}}{2}\mathop{\sum }\limits_{n=1}^{N-1}{\hat{{{{{{{{\boldsymbol{L}}}}}}}}}}_{n}^{2},$$

where H.C. denotes the Hermitian conjugate, *N* is the number of lattice sites with spacing *a*_*l*_, *g* is the gauge coupling, *m* is the fermion mass, ${\hat{\phi }}_{n}={\left({\hat{\phi }}_{n}^{1},{\hat{\phi }}_{n}^{2}\right)}^{T}$ is the staggered fermion field at site *n* with a red and a green component, and ${\hat{U}}_{n}$ is the gauge link connecting sites *n* and *n* + 1 (see Supplemental Information for the continuum model).

The last term in the Hamiltonian corresponds to the invariant Casimir operator of the theory and represents color electric field energy stored in the gauge links. Here, ${\hat{{{{{{{{\boldsymbol{L}}}}}}}}}}_{n}^{2}={\sum }_{a}{\hat{L}}_{n}^{a}{\hat{L}}_{n}^{a}={\sum }_{a}{\hat{R}}_{n}^{a}{\hat{R}}_{n}^{a}$ where ${\hat{L}}_{n}^{a}$ and ${\hat{R}}_{n}^{a}$ (with *a* = *x*, *y*, *z*) are respectively the left and right color electric field components on the link *n*. For a non-Abelian gauge group, the right and left color electric field are different and are related via the adjoint representation ${\hat{R}}_{n}^{a}={\sum }_{b}{({\hat{U}}_{n}^{{{{{\mathrm{adj}}}}}})}_{ab}{\hat{L}}_{n}^{b}$, where ${({\hat{U}}_{n}^{{{{{\mathrm{adj}}}}}})}_{ab}=2{{{{{{{\rm{Tr}}}}}}}}\left[{\hat{U}}_{n}{T}^{a}{\hat{U}}_{n}^{{{{\dagger}}} }{T}^{b}\right]$, *T*^*a*^ are the three generators of the SU(2) algebra and are given by half the Pauli matrices^41^.

### Symmetries and non-Abelian physics

By virtue of its gauge invariance, the Hamiltonian in Eq. (1) commutes with the local gauge transformation generators, also called the Gauss’s law operators, and are given by ${\hat{G}}_{n}^{a}\equiv {\hat{L}}_{n}^{a}-{\hat{R}}_{n-1}^{a}-{\hat{Q}}_{n}^{a},$ where the non-Abelian charges ${\hat{Q}}_{n}^{a}$ acting on the site *n* are defined as

2 $${\hat{Q}}_{n}^{a}=\mathop{\sum}\limits_{ij}{\hat{\phi }}_{n}^{i{{{\dagger}}} }{({T}^{a})}_{ij}{\hat{\phi }}_{n}^{j},\quad a=x,y,z.$$

More precisely, the physical Hilbert space of the theory is spanned by the eigenstates of the Gauss’s law operators ${\hat{G}}_{n}^{a}$. In the following, we choose to work in the sector with no external charges which is specified by ${\hat{{{{{{{{\boldsymbol{G}}}}}}}}}}_{n}\left|{{\Psi }}\right\rangle =0$, ∀ *n*, and in the neutral total charge sector ${\hat{Q}}_{\,{{{{\mathrm{tot}}}}}\,}^{a}\left|{{\Psi }}\right\rangle =\mathop{\sum }\nolimits_{n = 1}^{N}{\hat{Q}}_{n}^{a}\left|{{\Psi }}\right\rangle =0$, ∀ *a*.

Remarkably, the non-Abelian nature of the model allows the existence of gauge-invariant singlet states which are forbidden in the Abelian case due to symmetry constraints. To see this, we note that the total color charges ${\hat{Q}}_{\,{{{{\mathrm{tot}}}}}\,}^{a}=\mathop{\sum }\nolimits_{n = 1}^{N}{\hat{Q}}_{n}^{a}$ are conserved quantities and commute with the Hamiltonian. Besides the three non-Abelian charges, the Hamiltonian also commutes with the redness and greenness operators defined as $\hat{{{{{{{{\mathcal{R}}}}}}}}}=\mathop{\sum }\nolimits_{n = 1}^{N}{\hat{\phi }}_{n}^{1{{{\dagger}}} }{\hat{\phi }}_{n}^{1}-N/2$ and $\hat{{{{{{{{\mathcal{G}}}}}}}}}=\mathop{\sum }\nolimits_{n = 1}^{N}{\hat{\phi }}_{n}^{2{{{\dagger}}} }{\hat{\phi }}_{n}^{2}-N/2$, which respectively measure the red and green color charges. Because redness and greenness do not have convenient symmetry properties, it is more natural to use their difference (which is purely within the SU(2) gauge symmetry, since $\frac{\hat{{{{{{{{\mathcal{R}}}}}}}}}-\hat{{{{{{{{\mathcal{G}}}}}}}}}}{2}={\hat{Q}}_{\,{{{{\mathrm{tot}}}}}\,}^{z}$) and their sum (which is a global U(1) symmetry). We therefore define the baryon quantum number of the model as $\hat{B}=\frac{\hat{{{{{{{{\mathcal{R}}}}}}}}}+\hat{{{{{{{{\mathcal{G}}}}}}}}}}{2}=\frac{1}{2}\mathop{\sum }\nolimits_{n = 1}^{N}{\hat{\phi }}_{n}^{{{{\dagger}}} }{\hat{\phi }}_{n}-N/2$ which measures the matter-antimatter imbalance.

The existence of multiple conserved charges in the non-Abelian theory has to be contrasted with the Abelian U(1) case of quantum electrodynamics (QED), where the electric charge is the only conserved quantity. In QED, the total electric charge coincides with the baryon number *B* of the system^42^, and the neutral charge constraint thus imposes the value of the matter-antimatter imbalance to be zero. In other words, neutral gauge invariant states of QED must contain as many electrons as positrons leading to meson-type singlet states only. On the other hand, the constraint of neutral charge for the SU(2) theory ${\hat{Q}}_{\,{{{{\mathrm{tot}}}}}\,}^{i}\left|{{\Psi }}\right\rangle =0$, ∀ *i* does not enforce the value of the baryon quantum number *B*, since these are different quantum numbers. Therefore, it is possible to construct color-neutral gauge-invariant singlets with *B* ≠ 0, which are forbidden in QED. While the states in the *B* = 0 sector are similar to the neutral states of QED, the states in the sector with *B* ≠ 0 have no equivalent in Abelian theories. In particular, we will refer to the ground state in the sector with *B* = 1 as a baryon state, the ground state in *B* = 0 will be the vacuum and the first excited state will be called a meson state. A pictorial comparison of a meson and a baryon is given in Fig. 1b.

### Elimination of the gauge fields and qubit formulation

To study the energy spectrum of the SU(2) theory on a quantum computer, we map the lattice Hamiltonian in Eq. (1) to a qubit system. In one spatial dimension and with open boundary conditions, the gauge degrees of freedom can be integrated out and implicitly contribute to the non-Abelian physics through long-range exotic interactions^43–47^ (see Supplemental Note 2 for details and Eq. (6) below). This approach eliminates redundant degrees of freedom and allows us to calculate our target model with a minimal number of qubits. As a second step, a Jordan-Wigner transformation is applied to map the fermionic matter degrees of freedom to Pauli spin operators (see Supplementary Note 3 for details). The Hamiltonian is rescaled into the dimensionless form

3 $$\hat{H}=x\tilde{m}{\hat{H}}_{{{{{\mathrm{m}}}}}}+{\hat{H}}_{{{{{\mathrm{el}}}}}}+x{\hat{H}}_{{{{{\mathrm{kin}}}}}},$$

where we have defined the dimensionless Hamiltonian parameters $\tilde{m}={a}_{l}m$, $x=\frac{1}{{a}_{l}^{2}{g}^{2}}$, and we have added a constant to normalize the strong coupling (*x* → 0) ground state energy to zero. The different terms in the Hamiltonian are given by

4 $${\hat{H}}_{{{{{\mathrm{m}}}}}}=2\mathop{\sum }\limits_{n=1}^{N}\left(\frac{{(-1)}^{n}}{2}\left({\hat{\sigma }}_{2n-1}^{z}+{\hat{\sigma }}_{2n}^{z}\right)+1\right),$$

5 $${\hat{H}}_{{{{{\mathrm{kin}}}}}}=-\mathop{\sum }\limits_{n=1}^{N-1}\left({\hat{\sigma }}_{2n-1}^{+}{\hat{\sigma }}_{2n}^{z}{\hat{\sigma }}_{2n+1}^{-}+{\hat{\sigma }}_{2n}^{+}{\hat{\sigma }}_{2n+1}^{z}{\hat{\sigma }}_{2n+2}^{-}+\,{{{{\mathrm{H.C.}}}}}\,\right),$$

6 $${\hat{H}}_{{{{{\mathrm{el}}}}}}=	\, \frac{3}{8}\mathop{\sum }\limits_{n=1}^{N-1}(N-n)(1-{\hat{\sigma }}_{2n-1}^{z}{\hat{\sigma }}_{2n}^{z})\\ 	+\frac{1}{8}\mathop{\sum }\limits_{n=1}^{N-2}\mathop{\sum }\limits_{m > n}^{N-1}(N-m)\left({\hat{\sigma }}_{2n-1}^{z}-{\hat{\sigma }}_{2n}^{z}\right)\left({\hat{\sigma }}_{2m-1}^{z}-{\hat{\sigma }}_{2m}^{z}\right)\\ 	+\mathop{\sum }\limits_{n=1}^{N-2}\mathop{\sum }\limits_{m > n}^{N-1}(N-m)\left({\hat{\sigma }}_{2n-1}^{+}{\hat{\sigma }}_{2n}^{-}{\hat{\sigma }}_{2m}^{+}{\hat{\sigma }}_{2m-1}^{-}+{{{{{{{\rm{H.C.}}}}}}}}\right).$$

The Hamiltonian thus reduces to an effective qubit model with long-range interactions originating from the color electric field energy. In general, in order to calculate *N* staggered sites (i.e., *N*/2 physical sites), 2*N* qubits are necessary (Fig. 1a). The presence of non-diagonal interactions in Eq. (6) is a direct consequence of the non-Abelian character of the model (such terms are absent in the Abelian Schwinger model^11,41,42^). Note that even though the gauge degrees of freedom no longer appear explicitly in the Hamiltonian, their interaction with the matter fields is fully taken into account. In fact, gauge field observables such as the electric field density can be computed using the reduced Hamiltonian^42^ and are therefore still accessible to our quantum computation.

### Variational quantum search

**Fig. 2:**
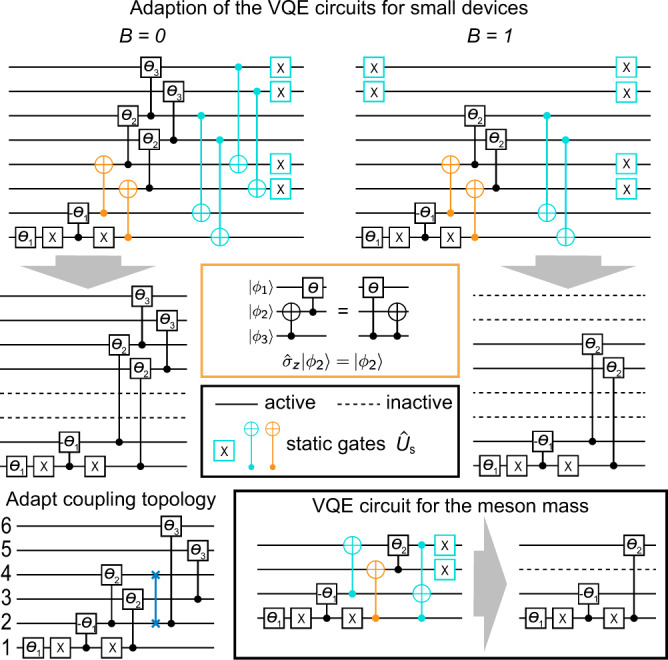
VQE ansatz circuits. The uppermost circuits for *N* = 4 can be reduced by absorbing the static colored gates into ${\hat{U}}_{s}$. The parametrized controlled gates are *Y*-rotations. For the orange gates, the circuit identity in the orange box has to be applied beforehand. This results in inactive qubits (dashed lines), which do not need to be physically available on the quantum device. Details of circuit reduction are discussed in Methods. In the lower left, the introduced SWAP gate for the adaptation to the architecture of the ibmq_casablanca processor is shown, with the qubit labeling as introduced in Fig. 3a (see below). The *N* = 2 circuits to estimate the meson mass are illustrated in the box in the bottom right.

To study the SU(2) baryon and meson states on current quantum computers, we employ the VQE approach to quantum calculations^48–50^, which consists of a classical optimizer that aims to minimize a cost function *C*(***θ***), where ***θ*** = (*θ*_1_, *θ*_2_, … ) are the variational parameters. The cost function is evaluated on quantum hardware, e.g., for the task of ground state preparation, we choose $C({{{{{{{\boldsymbol{\theta }}}}}}}})=\left\langle {{\Psi }}({{{{{{{\boldsymbol{\theta }}}}}}}})\right|\hat{H}\left|{{\Psi }}({{{{{{{\boldsymbol{\theta }}}}}}}})\right\rangle$ with an ansatz state $\left|{{\Psi }}({{{{{{{\boldsymbol{\theta }}}}}}}})\right\rangle =\hat{U}({{{{{{{\boldsymbol{\theta }}}}}}}})\left|{{{\Psi }}}_{0}\right\rangle$. $\left|{{{\Psi }}}_{0}\right\rangle$ represents a fiducial input state, and $\hat{U}({{{{{{{\boldsymbol{\theta }}}}}}}})$ a parameterized unitary evolution. In our case the circuits that implement such evolution are shown in Fig. 2. Our classical optimizer (see “Methods”) combines a mesh-based search with Bayesian optimisation techniques^51^, which avoids both costly gradient estimations and convergence in a local minimum. For each set of parameters ***θ*** we store the performed measurements of the multi-qubit Pauli operators contained in $\hat{H}$ (see “Methods” for a discussion about the decomposition of $\hat{H}$), which enables us to classically compute the corresponding value of *C*(***θ***) for different values of the Hamiltonian parameters.

To reduce the circuit depth and the number of qubits needed, i.e., to minimize error sources on the currently available NISQ devices, we exploit the freedom to split the circuit into two parts $\hat{U}({{{{{{{\boldsymbol{\theta }}}}}}}})={\hat{U}}_{s}{\hat{U}}^{\prime}({{{{{{{\boldsymbol{\theta }}}}}}}})$, where ${\hat{U}}_{s}$ contains static parts of the evolution that are not affected by the variational parameters, as shown in Fig. 2. Only the variational part of the circuit, ${\hat{U}}^{\prime}({{{{{{{\boldsymbol{\theta }}}}}}}})$ has to be carried out on quantum hardware, as part of the computation is relegated to classical preprocessing by transforming the Hamiltonian used in *C*(***θ***) as ${\hat{U}}_{s}^{{{{\dagger}}} }\hat{H}{\hat{U}}_{s}$. Generally this approach comes at the cost of increasing the number of Pauli operators that have to be measured. An additional practical advantage can be gained if this decomposition results—as in our case—in a separable state of active and inactive qubits, ${\hat{U}}^{\prime}({{{{{{{\boldsymbol{\theta }}}}}}}})\left|{{{\Psi }}}_{0}\right\rangle ={\hat{u}}_{a}({{{{{{{\boldsymbol{\theta }}}}}}}})\left|{{{\Psi }}}_{a}\right\rangle \otimes {\hat{u}}_{i}\left|{{{\Psi }}}_{i}\right\rangle$ whose second component can be efficiently computed classically. Hence, only the variational part of the ansatz state ${\hat{u}}_{a}({{{{{{{\boldsymbol{\theta }}}}}}}})\left|{{{\Psi }}}_{a}\right\rangle$ is implemented to measure the expectation value of the effective Hamiltonian acting on the active qubits $\left\langle {{{\Psi }}}_{i}\right|{\hat{u}}_{i}^{{{{\dagger}}} }{\hat{U}}_{s}^{{{{\dagger}}} }\hat{H}{\hat{U}}_{s}{\hat{u}}_{i}\left|{{{\Psi }}}_{i}\right\rangle$ (see “Methods” for more details).

### Preparation of the lightest baryon state on quantum hardware

Our VQE experiment determines the mass of the lightest baryon *M*_b_, which is defined as the gap between the energy of the lowest baryon state *E*_b_ and the vacuum state *E*_v_

7 $${M}_{{{{{\mathrm{b}}}}}}={E}_{{{{{\mathrm{b}}}}}}-{E}_{{{{{\mathrm{v}}}}}}.$$

As previously discussed, the lightest baryon state is the ground state of the Hamiltonian $\hat{H}$ given in Eq. (3) in the sector with baryon number *B* = 1, while the vacuum is the ground state in the sector with *B* = 0.

We experimentally prepare both states using the IBM Quantum Experience^37^ for a lattice with *N* = 4 spatial sites, $\tilde{m}=1$ and *x* ∈ [0, 5] (see “Methods” for a generalisation of our experimentally realized VQE scheme to larger lattices and parameter regimes). Since current quantum devices are restricted in the gate depth that can be faithfully implemented, we employ a problem-adapted efficient VQE circuit (Fig. 2) that creates a limited number of basis states and variationally combines them with adjustable weights. The circuit generates only color-neutral states in the *B* = 1 symmetry sector. Our implementation of the color symmetry and baryon number conservation is encoded directly into the VQE circuit and maintains the scalability of our algorithm. It differs from the pre-processing of the Hamiltonian by symmetry projections as discussed in ref. ^32^. We further reduce the explored state space by considering only basis elements that contain up to a total of four fermions and antifermions, which approximates the ground state well in the considered parameter range. In Methods, we propose a circuit that is not limited by the cut-off in the number of particles and valid for any Hamiltonian parameters, but comes at the expense of a higher gate depth (see Fig. 7 in “Methods”).

We apply the circuit-splitting technique explained above to our ansatz state, which reduces the number of qubits from eight to four (six) for the baryon (vacuum) state, as shown in Fig. 2. For our case the *B* = 1 baryon circuit can be seen as a specialized instance of the *B* = 0 circuit, so only the more general circuit needs to be implemented on the quantum hardware, namely the lower-left panel in Fig. 2.

**Fig. 3:**
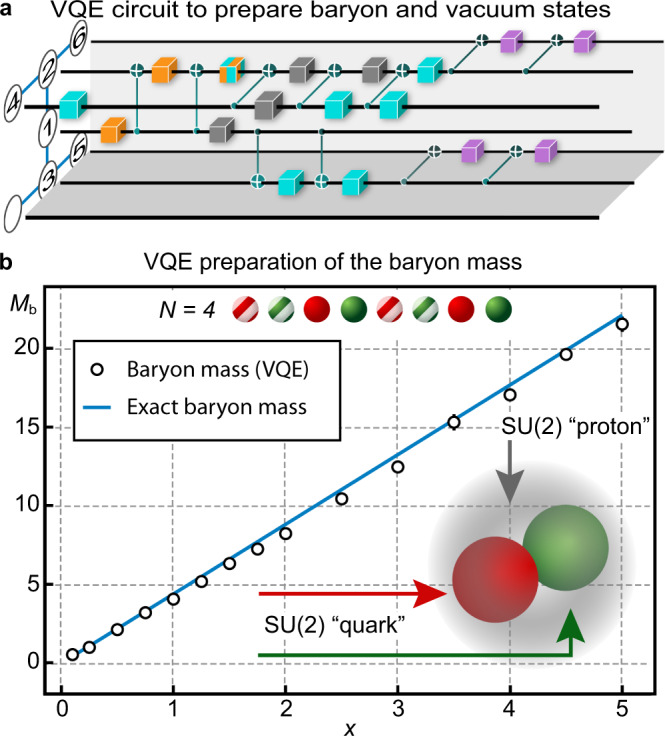
VQE calculation of a baryon. We variationally study an effective eight sites chain with the experimental circuit shown in (**a**). The boxes represent single-qubit gates. Gray boxes are fixed gates while the color coding indicates dependence from three variational parameters. Their exact implementation changes depending on the combination of the parameter values, which is automatically compiled from the original circuit shown in Fig. 2. This takes into account the coupling topology of the IBMQ Casablanca processor, which, together with the qubit identification for the *B* = 0 sector are shown on the left. **b** The circuit yields the mass of the baryon (error bars are smaller than markers, see “Methods” for a more detailed discussion), an SU(2)-"proton” (see inset), for a range of *x* and $\tilde{m}=1$ as explained in the main text.

The IBM Casablanca processor^37^ consists of seven qubits with the coupling topology displayed in Fig. 3a. We arranged the active qubits in a fashion such that only one SWAP gate is required to perform the circuit. The reduced circuit possesses three variational parameters, each modifying several single-qubit gates marked by the colored boxes in Fig. 3a. In order to perform one measurement of the Hamiltonian we need to repeat the ansatz state preparation and measure each of the 36 multi-qubit Pauli operators in which it is decomposed, and we average the measurement results over 8192 repetitions. In order to mitigate CNOT errors this procedure is repeated three times for different noise rates, which allows to extrapolate the results to the noise-free limit (see “Methods”).

The baryon mass obtained from the experimental VQE is shown in Fig. 3b and we find good agreement with the exact diagonalisation result.

### Accessing excited states on quantum hardware

As a next step in studying the properties of the baryon we address its mass ratio with its partner particle, the meson. We consider the lightest meson, which is the first excited state in the *B* = 0 sector with energy *E*_m_, and mass *M*_m_ = *E*_m_ − *E*_v_.

In order to access excited states within the VQE approach, we need to modify the cost function appropriately. Since the eigenstates of the Hamiltonian are mutually orthogonal, we add a term that penalizes variational states that overlap with the lower-energy eigenstates. More precisely, after obtaining the parameters ***θ***^*v*^ that minimize $\left\langle {{\Psi }}({{{{{{{\boldsymbol{\theta }}}}}}}})\right|\hat{H}\left|{{\Psi }}({{{{{{{\boldsymbol{\theta }}}}}}}})\right\rangle$, we consider as cost function $C({{{{{{{\boldsymbol{\theta }}}}}}}})=\left\langle {{\Psi }}({{{{{{{\boldsymbol{\theta }}}}}}}})\right|\hat{H}\left|{{\Psi }}({{{{{{{\boldsymbol{\theta }}}}}}}})\right\rangle +\beta | \langle {{\Psi }}({{{{{{{\boldsymbol{\theta }}}}}}}})| {{\Psi }}({{{{{{{{\boldsymbol{\theta }}}}}}}}}^{v})\rangle |$ to obtain the energy of the meson state, where *β* is a weight chosen larger than the expected energy gap^52^. The measurement of the overlap can be obtained by applying the unitary $\hat{U}{({{{{{{{\boldsymbol{\theta }}}}}}}})}^{{{{\dagger}}} }\hat{U}({{{{{{{{\boldsymbol{\theta }}}}}}}}}^{v})$ to the initial state. This composite unitary evolution can be realized by a further application of the inverse quantum circuit. Consequently, the overlap is directly given by the probability of measuring the initial state $\left|{{{\Psi }}}_{0}\right\rangle$ in the final state $\hat{U}{({{{{{{{\boldsymbol{\theta }}}}}}}})}^{{{{\dagger}}} }\hat{U}({{{{{{{{\boldsymbol{\theta }}}}}}}}}^{v})\left|{{{\Psi }}}_{0}\right\rangle$. This procedure is trivially extendable, i.e. higher excited states can be obtained recursively (see “Methods” for more details).

**Fig. 4:**
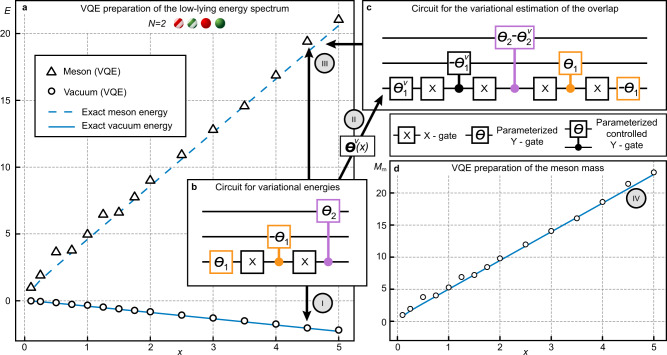
VQE calculation of the meson mass. To obtain the low-lying energy spectrum as shown in (**a**), we first employ the circuit in (**b**) to obtain the vacuum energy *E*_v_ (circles) in step I. Note that the employed gates are either rotations around the *y*-axis, the corresponding controlled gate, and bitflip *X*-gates. Subsequently in step II, the variational parameters minimizing *E*_v_ are used in the circuit in (**c**), which allows to estimate the overlap betweeen the ansatz state and the variational ground state (see main text and Methods). Together with the circuit **b** we perform a VQE calculation to obtain the first excited state energy *E*_m_ (step III, triangles). In the final step IV, we compute the energy difference *M*_m_ = *E*_m_ − *E*_v_ and obtain the mass of the meson, shown in panel **d**. In all panels, solid or dashed lines correspond to results derived via exact diagonalisation, error bars for experimental data are hidden due to the marker size (see “Methods” for a more detailed discussion).

Similar to the study of the baryon, we can simplify the VQE by enforcing the suitable symmetries of the state directly within the construction of the circuit, so that it creates only basis states that have the correct *B* number, are gauge singlets, and contain a limited number of particles. However, given the current limitations on the fidelities of available gates, calculating the required overlap is still a nontrivial task since it requires a deeper circuit. We, therefore, reduce our lattice size to enable the calculation on the quantum machine and compute the properties of the meson for *N* = 2. By applying the strategies discussed in the baryon case we can reduce the number of necessary qubits from four to three. In Fig. 4 we report the results from an experimental VQE calculation performed on the IBM Athens processor^38^, where we obtain the energies necessary to compute the meson mass. The vacuum and meson states are successfully computed with good accuracy (Fig. 4a), and the mass of the meson is shown in Fig. 4d. In Fig. 4b–c, we give the two circuits required to calculate the cost function for the computation of the excited meson state, namely one computing the expectation value of $\hat{H}$, and one computing the overlap with the previously calculated vacuum.

### Path towards the continuum limit

In the continuum limit, SU(2) gauge theory dictates that the masses of the baryon and the meson are equal^53^ because of a global SU(2) symmetry. Some lattice discretisations will preserve this degeneracy but for others it will only be restored in the continuum limit. Staggered fermions^40^, as used here, are in the latter category, which means the distinction between meson and baryon masses is a valuable measure of approaching the continuum limit.

**Fig. 5:**
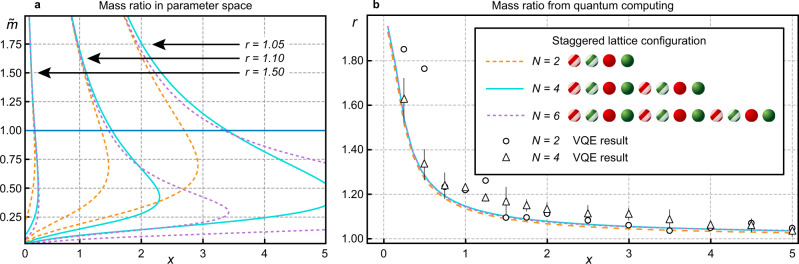
Mass ratio of lightest SU(2) meson and baryon in parameter space. **a** Displays lines of constant mass ratios *r* in the $(x,\tilde{m})$ plane obtained from exact diagonalisation for lattices of size *N* = 2, 4, 6. The blue horizontal line marks the cut shown in (**b**). For *N* = 2 we supply the experimental VQE results for the meson mass with data obtained via exact diagonalisation of the baryon energy, which is trivial for this lattice size. For *N* = 4 the meson energy is obtained via a classical calculation of a VQE including statistical errors, using the same measurement protocol as in the experimental run. Most of the error bars are hidden by the markers, see “Methods” for a more detailed discussion.

To study this effect quantitatively, let us define the hadron mass ratio

8 $$r=\frac{{M}_{{{{{\mathrm{m}}}}}}}{{M}_{{{{{\mathrm{b}}}}}}},$$

and obtain this quantity with explicit calculations from the qubit Hamiltonian in Eqs. (3–6) on classical computers. In general, in order to extrapolate lattice calculations to the continuum limit, it is necessary to take the limit *x* → *∞*, while keeping a physical length scale fixed^4^. Our small lattices are insufficient for performing a continuum extrapolation, but we are able to consider the *x* dependence while holding the mass ratio fixed. Figure 5a shows curves of constant *r* in the plane spanned by *x* and $\tilde{m}$. Notice that any curve with a fixed *r* > 1 does not allow for *x* → *∞*. The only constant-physics curve that allows it is the one in the limit of *r* → 1, therefore the correct value of the mass ratio in the continuum limit has to be 1 as required by the theory’s SU(2) global symmetry.

The large $\tilde{m}$ region of Fig. 5a, i.e., $\tilde{m} \; > \; 1$, is also insightful. Here the meson and baryon masses become dominated by the masses of the fermions and antifermions that they contain, relegating the meson-baryon mass difference arising from pair creation processes and gauge flux effects to be a small correction. This is reflected in Fig. 5a in two ways. One is that the curves of constant *r* become independent of *x* (getting more vertical toward the top of the graph). The other is that curves of constant *r* become independent of the lattice size *N*, since extended objects that probe the lattice boundaries always contain gauge flux which becomes a small effect at the top of the graph.

We perform experimental VQE calculations in the intermediate mass regime $\tilde{m}=1$ for several values of the gauge coupling within *x* ∈ [0, 5], marked by the horizontal blue line in Fig. 5a. The mass ratio *r* along this blue line is displayed in Fig. 5b for both the experimental VQE and exact diagonalisation, and the two methods show good agreement. The graph confirms that *r* approaches the value 1 for larger *x*, representing the correct restoration of mass degeneracy.

Furthermore, as is shown by comparing the exact diagonalisation data for *N* = 4 and *N* = 6, there is a clear indication that finite-size effects are quite limited already for small system sizes. We show in the Methods how our experimental VQE circuit for computing the baryon energy can be generalised to larger lattice sizes *N*, as required for the parameter range $\tilde{m}=1$, *x* ∈ [0, 5] that was discussed in Fig. 5b. We also extend our study to smaller fermion masses $\tilde{m}$ and provide a general circuit in the Methods that allows to compute the energy of the baryon for all parameter regimes. These extensions to our VQE experiments involve circuits beyond the capabilities of current quantum hardware and will require further experimental developments.

## Discussion

As of today, only Abelian physics and non-Abelian pure gauge theory have been simulated on a quantum computer. In this work we combine a quantum and a classical computer to prepare and study the hadrons of a non-Abelian gauge theory; this is made possible by the inclusion of dynamically coupled matter fields in the quantum computation.

While the gauge fields only appear implicitly in our approach, it is the gauge fields that provide the non-Abelian feature of the theory, i.e., the existence of a gauge-invariant baryon. This proof-of-concept demonstration was made possible by a resource-efficient approach for designing our VQE circuits. While necessary to alleviate the experimental requirements for implementing the full SU(2) gauge theory, this approach also paves the way for the development of future quantum simulators. In particular, the developed formalism offers a rigorous and systematic protocol to prepare highly entangled initial states for time evolution problems. We note that the theory developed in the first part of this paper lends itself to quantum simulations in other scientific areas and with other experimental platforms than the ones considered here. Finally, it is worth mentioning that the incorporation of digital-analog approaches^54^ in our VQE approach may be another promising direction to achieve a quantum advantage in practice.

Our work lays the foundation for a series of important next steps. Within the considered 1D SU(2) gauge theory, our work can be extended to study other hadrons including less familiar ones such as tetraquarks, with the goal of developing quantum simulators for nuclear physics. To this end, future work will include the extension to SU(3) gauge theory, since that is directly applicable to QCD. On this quest, extending our formulation to two and three spatial dimensions will have to be pursued, which can be done by following^55^. While the calculation of the low-lying spectrum of a non-Abelian model such as the one studied here can be performed with a classical computer, the methods developed in this benchmarking work offer the tools to tackle problems that are inherently difficult to address classically, such as the infamous sign problem which constitutes a well known issue in Monte Carlo methods for lattice gauge theories. In our approach, sign-problem afflicted models can be considered that include for example fermionic chemical potentials or topological terms, which both represent additions that can be included in the Hamiltonian formalism without any fundamental roadblock.

Ultimately, LGT calculations are indispensable for studying non-Abelian gauge theories, and a dramatic new breakthrough such as quantum computing has the potential to greatly extend the regime of numerical accessibility. Our computation of a complete non-Abelian benchmarking model, including both gauge and matter fields, represents an important first step and brings a path towards the quantum computation of non-Abelian LGT into view.

## Methods

### Hamiltonian decomposition and classical optimisation

The operators to be measured on the quantum hardware to estimate the expectation value $\langle \hat{H}\rangle$ can be readily obtained from Eqs. ((4)–(6)) in the main text after recalling that ${\hat{\sigma }}^{\pm }=({\hat{\sigma }}^{x}\pm i{\hat{\sigma }}^{y})/2$. In particular, the Hamiltonian $\hat{H}$ can be written as $\hat{H}=\mathop{\sum }\nolimits_{k = 1}^{n}{c}_{k}(x,\tilde{m}){\hat{P}}_{k}$, where ${c}_{k}(x,\tilde{m})$ is a real coefficient and ${\hat{P}}_{k}$ a 2*N*-qubit Pauli operator, e.g. ${\bigotimes }_{k = 1}^{2N}{\hat{\sigma }}_{k}^{z}$. For our Hamiltonian, we find by direct counting that the number of Pauli strings is given by *n* = 6*N*^2^ − 11*N* + 9 and grows quadratically in the number of qubits which is much smaller than the exponential upper bound of 4^2*N*^. Hence the value of $\langle \hat{H}\rangle$ is given by $\mathop{\sum }\nolimits_{k = 1}^{n}{c}_{k}\langle {\hat{P}}_{k}\rangle$, where we have omitted the dependence of *c*_*k*_ in *x* and $\tilde{m}$ for simplicity. In order to reduce the number of observables to measure, we form groups of commuting operators and measure only the operator with the lowest number of identity components out of each group. This allows to calculate the expectation value of the remaining operators in the same group employing only classical computations. Note that here we restrict to local measurements of the quantum state.

During the optimisation we consider different values of the Hamiltonian parameter *x* and it is clear that this only affects the weights ${c}_{k}(x,\tilde{m})$. Hence we can store the values of the $\langle {\hat{P}}_{k}\rangle$ obtained for different values of the variational parameters ***θ*** and supply our optimisation routine with the updated values of $\langle \hat{H}\rangle$ after a change of *x*. For the estimation of the baryon mass we make use of this fact by jointly measuring all operators ${\hat{P}}_{k}^{v}$ and ${\hat{P}}_{k}^{b}$ that are required for either the vacuum (*v*) or the baryon (*b*) energy respectively. This bears two advantages: first it allows to perform the error reduction described below, and second, it reduces the total number of calls that have to be made to the quantum processor.

Our optimisation routine employs an intertwined combination of a grid-based search (for exploration) and a Bayesian optimizer (for exploitation) that guide each other between subsequent iterations^51^. After enough refinements of the grid, it is hence guaranteed to find the global minimum. Since the optimizer accumulates more knowledge of the parameter space after each iteration and the measurements are independent of *x*, it is able to revisit any *x*-value to refine the optimisation after gaining these additional insights. On the other hand, the Bayesian techniques limit the number of optimisation parameters to around 20.

### Adaptations for NISQ hardware

In the following, we discuss the steps to adapt the quantum circuits to the currently available hardware in more detail. The simplifications proposed are general and not restricted to 1D systems and can in principle be applied to larger systems in higher dimension with dynamical gauge fields, as long as a qubit encoding is possible. As a concrete example, we consider the estimation of the baryon mass, where the circuits are constructed according to the targeted sector of the baryon number *B*, as outlined in the main text. Figure 2 in the main text focuses on the case *N* = 4 and illustrates the procedure formulated in the main text. For both baryon numbers we can rewrite the ansatz state as ${\hat{U}}_{s}{\hat{U}}^{\prime}({{{{{{{\boldsymbol{\theta }}}}}}}})\left|{{{\Psi }}}_{0}\right\rangle$, where we separate the trailing static part of the circuit which does not depend on the variational parameters and form the unitary ${\hat{U}}_{s}$. It becomes clear from $C({{{{{{{\boldsymbol{\theta }}}}}}}})=\left\langle {{{\Psi }}}_{0}\right|{\hat{U}}^{^{\prime} {{{\dagger}}} }({{{{{{{\boldsymbol{\theta }}}}}}}}){\hat{U}}_{s}^{{{{\dagger}}} }\hat{H}{\hat{U}}_{s}{\hat{U}}^{\prime}({{{{{{{\boldsymbol{\theta }}}}}}}})\left|{{{\Psi }}}_{0}\right\rangle$ that this corresponds to an effective transformation of the Hamiltonian $\hat{H}\,\mapsto {\hat{U}}_{s}^{{{{\dagger}}} }\hat{H}{\hat{U}}_{s}$ and an ansatz state produced with a shorter circuit ${\hat{U}}^{\prime}({{{{{{{\boldsymbol{\theta }}}}}}}})\left|{{{\Psi }}}_{0}\right\rangle$. Importantly, the transformation of $\hat{H}$ can be performed efficiently by applying a set of rules to the multi-qubit Pauli operators contained in it, e.g., a CNOT with control on qubit one maps ${\hat{\sigma }}_{x}^{2}{\hat{\sigma }}_{z}^{1}\,\mapsto -\!{\hat{\sigma }}_{y}^{2}{\hat{\sigma }}_{y}^{1}$. This transformation does not only reduce the depth of the circuit that needs to be implemented but, crucially, also reduces the required connectivity between the qubits employed in the experiment, which usually represents a major limiting factor, especially in superconducting architectures. Next, we note the circuit identity shown in the orange inset of Fig. 2, which allows to commute the two CNOT gates marked in orange with the controlled-*Y* rotations and enables us to absorb them into ${\hat{U}}_{s}$ as well. While this identity is generally not true, here the input state $\left|{\phi }_{2}\right\rangle$ is given by $\left|\downarrow \right\rangle$, which after the application of the CNOT results in a composite Bell-like state of the type $\sqrt{{p}_{\downarrow \downarrow }}\,\left|\downarrow \downarrow \right\rangle +\sqrt{{p}_{\uparrow \uparrow }}\,\left|\uparrow \uparrow \right\rangle$. Hence the control qubit for the following operation can be chosen arbitrarily among them.

In a second step, we eliminate inactive qubits from the circuit. Note that these are not ancilla qubits in the common notion, since they are still part of the encoded quantum state of the SU(2) theory; their entanglement with other qubits has rather been traded for additional measurements that have to be performed to estimate $\langle {\hat{U}}_{s}^{{{{\dagger}}} }\hat{H}{\hat{U}}_{s}\rangle$. Nevertheless, their quantum state is now separable from the active qubits, which allows to write the ansatz as ${\hat{U}}^{\prime}({{{{{{{\boldsymbol{\theta }}}}}}}})\left|{{{\Psi }}}_{0}\right\rangle ={\hat{u}}_{a}({{{{{{{\boldsymbol{\theta }}}}}}}})\left|{{{\Psi }}}_{a}\right\rangle \otimes {\hat{u}}_{i}\left|{{{\Psi }}}_{i}\right\rangle$, where ${\hat{u}}_{a}({{{{{{{\boldsymbol{\theta }}}}}}}})$ is the unitary containing the gates on the active qubits, while ${\hat{u}}_{i}$ corresponds to a static part that might be applied to the inactive qubits (we have ${\hat{u}}_{i}={{{{{{{\mathcal{I}}}}}}}}$ for all circuits employed here).

For example, in the case of *B* = 0, the qubits three and four are now inactive, and we can therefore modify the cost function as follows,

9 $$C({{{{{{{\boldsymbol{\theta }}}}}}}})=\left\langle {{{\Psi }}}_{a}\right|{\hat{u}}_{a}^{{{{\dagger}}} }({{{{{{{\boldsymbol{\theta }}}}}}}})\left[{\left\langle \downarrow \downarrow \right|}_{34}{\hat{U}}_{s}^{{{{\dagger}}} }\hat{H}{\hat{U}}_{s}{\left|\downarrow \downarrow \right\rangle }_{34}\right]{\hat{u}}_{a}({{{{{{{\boldsymbol{\theta }}}}}}}})\left|{{{\Psi }}}_{a}\right\rangle ,$$

where the term in the square brackets is an operator in the Hilbert space of the remaining qubits one, two and five to eight. After relabeling, we arrive at the six qubit circuit in Fig. 2. A similar procedure is performed for the circuit designed for the sector *B* = 1 with the addition that also the qubits seven and eight can be removed, i.e. the effective Hamiltonian in the brackets reads $\left[{\left\langle \downarrow \downarrow \downarrow \downarrow \right|}_{3478}{\hat{U}}_{s}^{{{{\dagger}}} }\hat{H}{\hat{U}}_{s}{\left|\downarrow \downarrow \downarrow \downarrow \right\rangle }_{3478}\right]$, which leaves the corresponding circuit consisting of four qubits.

We remark that the six qubit circuit can be employed in both cases, if the qubits are correctly relabeled and we only remove the qubits seven and eight in the *B* = 1 case. Since we are interested in the difference of the two eigenenergies, the latter approach is able to remove erroneous admixtures to the quantum state, since the estimations of the energies are calculated from the same sample. In more detail, for any observable $\hat{O}$ to be measured, we have $\langle \hat{O}\rangle =(1-{p}_{e}){{{{{{{\rm{Tr}}}}}}}}[\hat{O}\hat{\rho }({{{{{{{\boldsymbol{\theta }}}}}}}})]+{p}_{e}{{{{{{{\rm{Tr}}}}}}}}[\hat{O}{\hat{\rho }}_{e}]$, if we assume that at least some systematic, ***θ***-independent part of the errors can be modeled by a convex combination to the density matrix with error probability *p*_*e*_. Then ${\langle \hat{O}\rangle }_{B = 1}-{\langle \hat{O}\rangle }_{B = 0}=(1-{p}_{e})\left({{{{{{{\rm{Tr}}}}}}}}[\hat{O}\hat{\rho }({{{{{{{{\boldsymbol{\theta }}}}}}}}}_{B = 1})]-{{{{{{{\rm{Tr}}}}}}}}[\hat{O}\hat{\rho }({{{{{{{{\boldsymbol{\theta }}}}}}}}}_{B = 0})]\right)$ is independent of ${\hat{\rho }}_{e}$.

### VQE computation of the meson mass

In this section we detail the experimental VQE protocol for the quantum simulation of the meson mass *E*_m_ − *E*_v_. The energies *E*_v_ and *E*_m_ are found as the energies of the ground and first excited state in the *B* = 0 subsector respectively. The full circuit to estimate the ground state energy for *N* = 2 is shown in the lower right of Fig. 2 in the main text. We reduce the circuit to three qubits by employing the methods described above. In the main text, we explain the protocol to access the first excited state of the sector, here we cover the calculation of the overlap in more detail. The VQE result of the ground state (i.e., vacuum state) for a specific value of *x* entails the parameters ***θ***^*v*^, such that $\hat{U}({{{{{{{{\boldsymbol{\theta }}}}}}}}}^{v})\left|{{{\Psi }}}_{0}\right\rangle$ is the ground state of $\hat{H}$ found through the quantum simulation. The overlap with any other state generated by a new set of variational parameters is given by $| \left\langle {{{\Psi }}}_{0}\right|{\hat{U}}^{{{{\dagger}}} }({{{{{{{\boldsymbol{\theta }}}}}}}})\hat{U}({{{{{{{{\boldsymbol{\theta }}}}}}}}}^{v})\left|{{{\Psi }}}_{0}\right\rangle {| }^{2}$. We can access the Hermitian conjugate of $\hat{U}({{{{{{{\boldsymbol{\theta }}}}}}}})$ by applying the inverse circuit, i.e., the reversed gate sequence with all parameters multiplied by −1. Since $\left|{{{\Psi }}}_{0}\right\rangle ={\left|\downarrow \right\rangle }^{\otimes 2N}$ is an element of the computational basis (the eigenstates of ${\bigotimes }_{k = 1}^{2N}{\hat{\sigma }}_{k}^{z}$), we can obtain the required overlap as the probability of measuring the state ${\hat{U}}^{{{{\dagger}}} }({{{{{{{\boldsymbol{\theta }}}}}}}})\hat{U}({{{{{{{{\boldsymbol{\theta }}}}}}}}}^{v})\left|{{{\Psi }}}_{0}\right\rangle$ in the computational basis and obtaining the initial state $\left|{{{\Psi }}}_{0}\right\rangle$. Note that due to the approximately doubled circuit depth, the calculation is much more susceptible to gate errors and environmental noise processes, which can render the measurement of the overlap experimentally infeasible.

As an alternative method to obtain the first excited state, we perform a variational search in the space orthogonal to the variational ground state $\hat{U}({{{{{{{{\boldsymbol{\theta }}}}}}}}}^{v})\left|{{{\Psi }}}_{0}\right\rangle$ by implementing a Gram-Schmidt orthogonalisation procedure. The ansatz for the first excited state is thus searched in the form

10 $$\left|{{{\Psi }}}_{1}({{{{{{{\boldsymbol{\theta }}}}}}}})\right\rangle ={{{{{{{{\mathcal{N}}}}}}}}}_{0}\left(\hat{U}({{{{{{{\boldsymbol{\theta }}}}}}}})-\left\langle {{{\Psi }}}_{0}\right|{\hat{U}}^{{{{\dagger}}} }({{{{{{{{\boldsymbol{\theta }}}}}}}}}^{v})\hat{U}({{{{{{{\boldsymbol{\theta }}}}}}}})\left|{{{\Psi }}}_{0}\right\rangle \hat{U}({{{{{{{{\boldsymbol{\theta }}}}}}}}}^{v})\right)\left|{{{\Psi }}}_{0}\right\rangle ,$$

with the normalisation factor given by

11 $${{{{{{{{\mathcal{N}}}}}}}}}_{0}={\left(1-| \left\langle {{{\Psi }}}_{0}\right|{\hat{U}}^{{{{\dagger}}} }({{{{{{{{\boldsymbol{\theta }}}}}}}}}^{v})\hat{U}({{{{{{{\boldsymbol{\theta }}}}}}}})\left|{{{\Psi }}}_{0}\right\rangle {| }^{2}\right)}^{-1/2}.$$

The new energy cost function to minimize $C({{{{{{{\boldsymbol{\theta }}}}}}}})=\left\langle {{{\Psi }}}_{1}({{{{{{{\boldsymbol{\theta }}}}}}}})\right|\hat{H}\left|{{{\Psi }}}_{1}({{{{{{{\boldsymbol{\theta }}}}}}}})\right\rangle$ considers only components of the variational state orthogonal to the ground state, and explicitly reads

12 $$C({{{{{{{\boldsymbol{\theta }}}}}}}})=\frac{\left\langle {{\Psi }}({{{{{{{\boldsymbol{\theta }}}}}}}})\right|\hat{H}\left|{{\Psi }}({{{{{{{\boldsymbol{\theta }}}}}}}})\right\rangle -{E}_{{{{{\mathrm{v}}}}}}| \left\langle {{{\Psi }}}_{0}\right|{\hat{U}}^{{{{\dagger}}} }({{{{{{{{\boldsymbol{\theta }}}}}}}}}^{v})\hat{U}({{{{{{{\boldsymbol{\theta }}}}}}}})\left|{{{\Psi }}}_{0}\right\rangle {| }^{2}}{1-| \left\langle {{{\Psi }}}_{0}\right|{\hat{U}}^{{{{\dagger}}} }({{{{{{{{\boldsymbol{\theta }}}}}}}}}^{v})\hat{U}({{{{{{{\boldsymbol{\theta }}}}}}}})\left|{{{\Psi }}}_{0}\right\rangle {| }^{2}}.$$

This has the advantage of obtaining the excited state energy, even if the quantum circuit can only produce small components of the excited state. However, the dependence on the ground state energy *E*_v_ demands a precise estimate of the latter, since the structure of the denominator implies vast distortions due to mistakes in *E*_v_ when the overlap is estimated to be close to one. Hence we resort to the cost function described in the main text for the experimental calculation of the meson energy.

### Implementation on the IBM processors

All reduced circuits can be straightforwardly implemented on hardware that offers qubits arranged in a simple chain with nearest-neighbor coupling, since all further connectivity requirements have been mitigated into measurements of the effective Hamiltonian $\left\langle {{{\Psi }}}_{i}\right|{\hat{U}}_{s}^{{{{\dagger}}} }\hat{H}{\hat{U}}_{s}\left|{{{\Psi }}}_{i}\right\rangle$ (recall ${\hat{u}}_{i}={{{{{{{\mathcal{I}}}}}}}}$ here). We implement the six-qubit circuit for the baryon mass on the seven-qubit ibmq_casablanca processor, which possesses the coupling map shown in Fig. 3b in the main text and hence requires at least one SWAP operation. We modify the circuit as shown in Fig. 2 in the main text and relabel the operators in the effective Hamiltonian, such that the SWAP has not to be reversed.

During the experiment, the main sources of error are the statistical readout, imperfect application of the CNOT gates, and readout errors, i.e., a false assignment of basis states during the measurement process. An estimate of the statistical error can be done by standard techniques employing the sample mean and sample covariance. Note that such error does not quantify a deviation to the true expectation value of $\hat{H}$ with respect to the variational state, since the measurement is performed via subsequent measurements of sets containing the Pauli-operators spanning $\hat{H}$. Furthermore, each call to the quantum processor entails a set of calibrating circuits to mitigate readout errors, i.e. allow one to estimate the map Λ that mixes the true measurement probabilities ***p***_true_ into the observed ones ***p***_obs_ = Λ ***p***_true_, such that one obtains an estimate of the true probabilities by an inversion of the map. Note that no contribution to the final error bar is obtained via that procedure. To include and minimize the error stemming from the entangling gates, we employ an extrapolation of the CNOT errors to mitigate their effect (the final error bars do not include uncertainty resulting from this inversion). We replace each CNOT in the circuits by either three or five CNOT gates to artificially enhance the effect of the introduced error. The errors are small enough that the experimental points with different numbers of CNOT gates can be linearly interpolated without needing any higher-order correction to give the result in the hypothetical case of vanishing CNOT error^56,57^. The latter procedure takes into account the statistical error influencing each of the results, hence an error is associated to the linear fit, which is the one we show in the figures of the main text. Let us remark however that the data collection has been performed over multiple calibration cycles automatically performed by IBM which can have non-tractable influence on the shown error bars.

### Extension for future quantum computers

As explained in the previous sections, our results presented in the main text for the baryon and the meson rely on carefully chosen measures such as the mass cut-off and circuit-splitting technique. These measures are necessary given the current technological status and restrictions imposed by NISQ devices. It is however important to present tools for the investigation of our model in light of foreseeable more powerful quantum hardware. Future quantum computers will offer the possibility to use more qubits with a higher level of qubit control and increased circuit depth. To address the advantages and potential offered by these future quantum computers, we have designed circuits free of the measures taken in the case of NISQ devices. To be more precise, we first show that the VQE approach applied in our experiment can be extended to larger lattice sizes and any parameter regime by emulating the VQE protocol on a classical device, where we estimate the mass of the baryon for *N* = 6 spatial sites (12 qubits). The calculation involves a circuit that does not limit the number of particles that are contained in the states that are generated, but comes at the expense of a high gate depth. To alleviate the experimental requirements, we also propose an alternative circuit for the baryon in the case of *N* = 4, which allows us to obtain the baryon state in all parameter regimes with high fidelity.

As a proof of principle, we perform numerical simulations of a VQE protocol employing a generalized ansatz circuit to estimate the baryon mass for a spatial lattice of six sites (*N* = 6, 12 qubits). Here, we do not take the statistical quantum measurement noise into account. The ansatz we choose is the following. Given a baryon number *B*, we employ the ground state at *x* → 0 (strong coupling) as our input $\left|{{{\Psi }}}_{0}\right\rangle$. For *B* = 1 this corresponds to a red-green particle pair at the spatial site *N* = 6 and in the case *B* = 0 to the bare vacuum state. Note that for *B* = 1, the ground state for *x* → 0 is *N*/2-fold degenerate, corresponding to the possible number of sites the red-green particle pair could occupy. For small, finite values of the parameter *x*, the kinetic term in our Hamiltonian (see Eq. (5) in the main text) lifts the degeneracy and a second-order perturbation expansion shows that the state corresponding to the red-green particle pair at *N*-th spatial site of the chain has the lowest energy, which motivates the choice of our initial state.

The variational circuit consists of layers of pairwise, excitation-preserving gates between neighboring qubits, i.e., the unitary of the *k*-th layer reads

13 $${\hat{{{{{{{{\mathcal{U}}}}}}}}}}_{k}=\mathop{\sum }\limits_{j=1}^{2N-1}{\hat{U}}_{j,j+1}({\theta }_{j,k}).$$

**Fig. 6:**
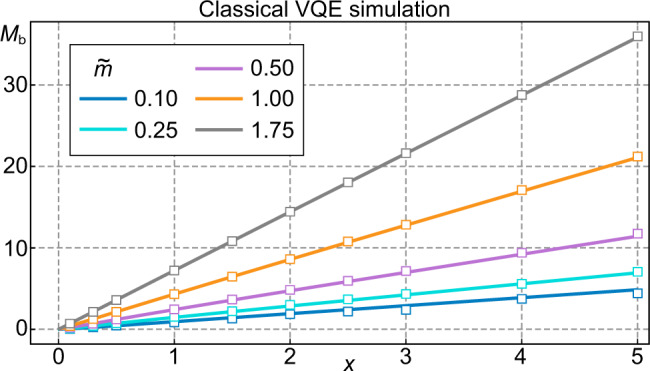
Classical simulation of a VQE to estimate the baryon mass for *N* = 6. For different values of $\tilde{m}$ we calculate the mass either via an exact diagonalisation (solid lines) or with the magnetisation preserving VQE ansatz in equation (13) (boxes). The case *N* = 4 and $\tilde{m}=1$ calculated on real quantum hardware is shown in Fig. 3 of the main text.

Here, the unitaries ${\hat{U}}_{j,j+1}(\theta )$ are given by parameterized SWAP gates. Note that each ${\hat{{{{{{{{\mathcal{U}}}}}}}}}}_{k}$ preserves the total spin $\langle {\hat{\sigma }}_{\,{{{{\mathrm{tot}}}}}\,}^{z}\rangle$ and hence ensures that the final state also lies in the chosen subspace characterized by *B*. In fact, it can be easily shown that the baryon number in qubit formulation is given by $\hat{B}=\frac{{\hat{\sigma }}_{\,{{{{\mathrm{tot}}}}}\,}^{z}}{4}$, and therefore subspaces with fixed baryon number correspond to subspaces with fixed total magnetisation. Employing 10 (15) layers in the *B* = 0 (*B* = 1) sector, we obtain the baryon mass shown in Fig. 6 for different values of $\tilde{m}$. Importantly, this procedure grants access to the whole parameter space illustrated in Fig. 5 of the main text.

In general, for *N* sites, we have 2*N* − 1 variational parameter per layer of gates. Therefore, for larger lattices, the use of Bayesian optimizer will be limited by the increase in the number of variational parameters. However other optimization methods are being developed to overcome this issue^58^. The high depth of the circuit required by this method can also be subject to scaling issues, but the development of more efficient circuits, that can be designed employing expressivity analysis^59^, error mitigation techniques^60^, and experimental advancements will bring new possibilities into view.

In order to further lower the computational effort of the previous brute force approach, we propose another solution that reduces the depth of the circuit and number of variational parameters. We consider a circuit that generates the ansatz state for the lightest baryon on a lattice with *N* = 4 sites and arbitrary Hamiltonian parameters.

In Table 1, we have listed all the basis states whose total magnetisation is equal to 4 (corresponding to a baryon quantum number *B* = 1), and are annihilated by ${\hat{Q}}_{{{{{{{{\rm{tot}}}}}}}}}^{z}$. There are 16 such states and 12 of them must be combined pairwise in order to form color singlet combinations, i.e. such that an application of the three non-Abelian charges ${\hat{Q}}_{{{{{{{{\rm{tot}}}}}}}}}^{a}$ with *a* = *x*, *y*, *z* is equal to zero. Basis states which have to be combined are written with the same numeric index and located in the same row of Table 1. For instance, the two states appearing in the fifth row must be combined in the following way

14 $$\left|{\tilde{s}}_{5}\right\rangle =\frac{1}{\sqrt{2}}\left(\left|{s}_{5}\right\rangle -\left|{s}_{5}^{\prime}\right\rangle \right)$$

to be a common eigenstate of the three non-Abelian charges with eigenvalue zero (for more details, see Supplementary Note 4). For larger lattice sizes *N*, the construction of the singlet states becomes more involved and will not be addressed here.

**Table 1:** All basis states with baryon number *B* = 1 for a lattice with *N* = 4 spatial sites.

*N* = 4, *B* = 1 basis states
$$\left|{s}_{1}\right\rangle =\left|\uparrow \uparrow \,\uparrow \uparrow \,\uparrow \uparrow \,\downarrow \downarrow \right\rangle$$
$$\left|{s}_{2}\right\rangle =\left|\uparrow \uparrow \,\uparrow \uparrow \,\downarrow \downarrow \,\uparrow \uparrow \right\rangle$$
$$\left|{s}_{3}\right\rangle =\left|\uparrow \uparrow \,\downarrow \downarrow \,\uparrow \uparrow \,\uparrow \uparrow \right\rangle$$
$$\left|{s}_{4}\right\rangle =\left|\downarrow \downarrow \,\uparrow \uparrow \,\uparrow \uparrow \,\uparrow \uparrow \right\rangle$$
$$\left|{s}_{5}\right\rangle =\left|\uparrow \downarrow \,\downarrow \uparrow \,\uparrow \uparrow \,\uparrow \uparrow \right\rangle$$
$$\left|{s}_{5}^{\prime}\right\rangle =\left|\downarrow \uparrow \,\uparrow \downarrow \,\uparrow \uparrow \,\uparrow \uparrow \right\rangle$$
$$\left|{s}_{6}\right\rangle =\left|\uparrow \downarrow \,\uparrow \uparrow \,\downarrow \uparrow \,\uparrow \uparrow \right\rangle$$
$$\left|{s}_{6}^{\prime}\right\rangle =\left|\downarrow \uparrow \,\uparrow \uparrow \,\uparrow \downarrow \,\uparrow \uparrow \right\rangle$$
$$\left|{s}_{7}\right\rangle =\left|\uparrow \downarrow \,\uparrow \uparrow \,\uparrow \uparrow \,\downarrow \uparrow \right\rangle$$
$$\left|{s}_{7}^{\prime}\right\rangle =\left|\downarrow \uparrow \,\uparrow \uparrow \,\uparrow \uparrow \,\uparrow \downarrow \right\rangle$$
$$\left|{s}_{8}\right\rangle =\left|\uparrow \uparrow \,\uparrow \uparrow \,\uparrow \downarrow \,\downarrow \uparrow \right\rangle$$
$$\left|{s}_{8}^{\prime}\right\rangle =\left|\uparrow \uparrow \uparrow \uparrow \,\downarrow \uparrow \,\uparrow \downarrow \right\rangle$$
$$\left|{s}_{9}\right\rangle =\left|\uparrow \uparrow \,\uparrow \downarrow \,\uparrow \uparrow \,\downarrow \uparrow \right\rangle$$
$$\left|{s}_{9}^{\prime}\right\rangle =\left|\uparrow \uparrow \,\downarrow \uparrow \,\uparrow \uparrow \,\uparrow \downarrow \right\rangle$$
$$\left|{s}_{10}\right\rangle =\left|\uparrow \uparrow \,\uparrow \downarrow \,\downarrow \uparrow \,\uparrow \uparrow \right\rangle$$
$$\left|{s}_{10}^{\prime}\right\rangle =\left|\uparrow \uparrow \,\downarrow \uparrow \,\uparrow \downarrow \,\uparrow \uparrow \right\rangle$$

States in the same row must be combined together to form a color singlet as exemplified in Eq. (14).

**Fig. 7:**
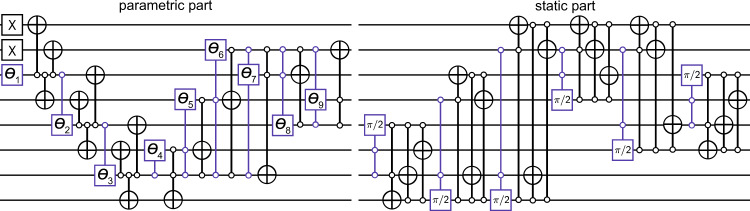
General baryon circuit for *N* = 4. The parametric part of the circuit involves nine variational parameters (*θ*_1_, *θ*_2_, …, *θ*_9_), while the static part can be incorporated into the Hamiltonian to reduce the computational effort as discussed in the main text. The colored gates mark (controlled) rotations around the *y*-axis with the angle of rotation indicated. White control marks denote the active application of the gate when the control is in $\left|\downarrow \right\rangle$. The circuit, when applied to the initial state $\left|\uparrow \uparrow \,\uparrow \uparrow \,\uparrow \uparrow \,\uparrow \uparrow \right\rangle$, generates the 16 basis states satisfying the *B* = 1 symmetry (reported in Table 1) and combines them to form color singlet thus reducing the total number of necessary variational parameters. Classical simulations of noiseless VQE using this circuit have demonstrated a high fidelity with the exact ground state in the *B* = 1 sector.

Once we have constructed a basis for the *B* = 1 symmetry sector composed of color singlet states, we can parametrize an ansatz for the lightest baryon considering a superposition of such basis elements with real coefficients. For example we can use hyperspherical coordinates and consider

15 $$\left|{{\Psi }}({{{{{{{\boldsymbol{\theta }}}}}}}})\right\rangle =\mathop{\sum }\limits_{n=1}^{10}{a}_{n}({{{{{{{\boldsymbol{\theta }}}}}}}})\left|{\tilde{s}}_{n}\right\rangle ,$$

where $\left|{\tilde{s}}_{n}\right\rangle =(\left|{s}_{n}\right\rangle -\left|{s}_{n}^{\prime}\right\rangle )/\sqrt{2}$ are the color singlet combinations of basis states appearing in Table 1, ${a}_{n}({{{{{{{\boldsymbol{\theta }}}}}}}})=\mathop{\prod }\nolimits_{i = 1}^{n-1}\sin ({\theta }_{i})\cos ({\theta }_{n})$ for *n* = 1, 2, …, 9, ${a}_{10}({{{{{{{\boldsymbol{\theta }}}}}}}})=\mathop{\prod }\nolimits_{i = 1}^{9}\sin ({\theta }_{i})$, and ***θ*** = (*θ*_1_, *θ*_2_…*θ*_9_) is a vector of nine variational parameters. Note that only nine parameters are required to describe the ansatz state since the tenth is automatically fixed by the normalisation. The circuit generating the ansatz state is represented in Fig. 7 and has been separated into two parts. The first parametric part contains the nine variational parameters and creates the following superposition

16 $$\left|\psi ({{{{{{{\boldsymbol{\theta }}}}}}}})\right\rangle ={\hat{U}}^{\prime}({{{{{{{\boldsymbol{\theta }}}}}}}})\left|\uparrow \uparrow \,\uparrow \uparrow \,\uparrow \uparrow \,\uparrow \uparrow \right\rangle =\mathop{\sum }\limits_{n=1}^{10}{a}_{n}({{{{{{{\boldsymbol{\theta }}}}}}}})\left|{s}_{n}\right\rangle$$

where ${\hat{U}}^{\prime}({{{{{{{\boldsymbol{\theta }}}}}}}})$ is the unitary representing the parametric part of the circuit, $\left|\uparrow \uparrow \,\uparrow \uparrow \,\uparrow \uparrow \,\uparrow \uparrow \right\rangle$ is the input state and $\left|{s}_{n}\right\rangle$ are the basis states in Table 1. The purpose of the second part of the circuit is to impose the color symmetry and hence to produce the color symmetric superpositions as in Eq. (15), i.e., $\left|{{\Psi }}({{{{{{{\boldsymbol{\theta }}}}}}}})\right\rangle ={\hat{U}}_{s}\left|\psi ({{{{{{{\boldsymbol{\theta }}}}}}}})\right\rangle ={\hat{U}}_{s}{\hat{U}}^{\prime}({{{{{{{\boldsymbol{\theta }}}}}}}})\left|\uparrow \uparrow \,\uparrow \uparrow \,\uparrow \uparrow \,\uparrow \uparrow \right\rangle$ with ${\hat{U}}_{s}$ the unitary representing the static part of the circuit in Fig. 7. Let us note that the static part of the circuit possesses a block structure with an elementary block made of a double controlled *π*/2*Y*-rotation followed by three Toffoli gates. There are six elementary blocks corresponding to the six states which need to be combined in Table 1. As an example, let us consider the first block and see its action on the state $\left|\psi ({{{{{{{\boldsymbol{\theta }}}}}}}})\right\rangle$. The controlled rotation acts only on the basis state $\left|{s}_{5}\right\rangle$, which is the only one having both spins pointing down at positions two and three, and hence generates the state $\left|\uparrow \downarrow \,\downarrow \downarrow \,\uparrow \uparrow \,\uparrow \uparrow \right\rangle$ with weight −*a*_5_(***θ***). The three subsequent Toffoli gates transform this state into $\left|{s}_{5}^{\prime}\right\rangle$, resulting in the color singlet state given in Eq. (14). The other blocks act similarly, and after the application of the static part, we obtain the ansatz state given in Eq. (15). Also note that the overall structure of the circuit would in principle allow the use of the splitting technique to further reduce the computational effort as described in the previous sections. Classical simulations of noise-free VQE with this circuit have demonstrated high fidelity with the exact ground state in the *B* = 1 sector and for any value of the Hamiltonian parameters.

## Supplementary information

Supplementary information

## Data Availability

Source data are provided with the manuscript. Source data are provided with this paper.

## Source data

Source Data
