# Supplementary material for: SU(2) hadrons on a quantum computer via a variational approach
Source: Nat Commun. 2021 Nov 11;12:6499. doi: 10.1038/s41467-021-26825-4 (PMC8586147; doi:10.1038/s41467-021-26825-4)
Supplement: Supplementary file 1 — Supplementary information [file 41467_2021_26825_MOESM1_ESM.pdf]

Supplementary Information: SU(2) hadrons on a quantum computer via a variational approach

Yasar Atas *,[1,2,†] Jinglei Zhang *,[1,2,‡] Randy Lewis,[3] Amin Jahanpour,[1,2] Jan F. Haase,[1,2,4,§] and Christine A. Muschik[1,2,5]

[1]*Institute for Quantum Computing, University of Waterloo, Waterloo, ON, Canada, N2L 3G1*
[2]*Department of Physics & Astronomy, University of Waterloo, Waterloo, ON, Canada, N2L 3G1*
[3]*Department of Physics and Astronomy, York University, Toronto, ON, Canada, M3J 1P3*
[4]*Institut für Theoretische Physik und IQST, Universität Ulm, Albert-Einstein-Allee 11, D-89069 Ulm, Germany*
[5]*Perimeter Institute for Theoretical Physics, Waterloo, ON, Canada, N2L 2Y5*

(Dated: September 16, 2021)

Supplementary Note 1. SU(2) gauge theory in the continuum

In the following, we consider the continuum version of the SU(2) Yang-Mills Hamiltonian and show how its discretisation leads to the Kogut-Susskind Hamiltonian given in the main text. We regard the non-Abelian SU(2) Yang-Mills model describing fermions and antifermions interacting via color electric fields in one spatial dimension. In this model, the fermions and antifermions carry a color charge which we will refer to as red and green. The interactions between the fermions are mediated by the gauge field or "color electric field". We denote the gauge field vector potential at position z with temporal and spatial components $\hat{A}_0^a(z)T^a$ and $\hat{A}_1^a(z)T^a$ respectively, where $T^a = \sigma^a/2$ are the three generators of the SU(2) Lie algebra, and σ^a the a-th Pauli matrix ($a = x, y, z$). From now on, we adopt the temporal or Weyl gauge $\hat{A}_0^a(z) = 0$ and Einstein's summation convention will apply on repeating indices in color space, but not on lattice indices. The color electric field also carries a group index a and is given by $\hat{L}^a(z) = -\partial_t \hat{A}_1^a(z)$. As the canonical conjugate momentum of $\hat{A}_1^a(z)$, the color electric field satisfies $[\hat{A}_1^a(z), \hat{L}^b(y)] = i\delta_{ab}\delta(z-y)$. In the continuum, the Yang-Mills Hamiltonian is given by [1]

$$\hat{H}_{\text{cont}} = \int \mathrm{d}z \left[\hat{\bar{\psi}}(z)\gamma^1 \left(-i\partial_z + g\hat{A}_1^a(z)T^a\right)\hat{\psi}(z) + m\hat{\bar{\psi}}(z)\hat{\psi}(z) + \frac{1}{2}\hat{L}^a(z)\hat{L}^a(z)\right], \tag{1}$$

where $\hat{\psi}(z) = (\hat{\psi}_1(z), \hat{\psi}_2(z))^T$ is a two-component spinor representing the matter fields. The fermion mass is denoted by m, and g quantifies the matter-field coupling constant, while γ^μ are the Dirac matrices satisfying the anticommutation relations $\{\gamma^\mu, \gamma^\nu\} = 2\eta^{\mu\nu}$ with $\eta = \mathrm{diag}(1, -1)$ the metric tensor. We further use the shorthand notation $\hat{\bar{\psi}}(z) = \hat{\psi}^\dagger(z)\gamma^0$. In one dimension, a convenient representation for the Dirac matrices is given by $\gamma^0 = \sigma^z$ and $\gamma^1 = i\sigma^y$. Note that the first term in Supplementary Eq. (1) represents the gauge-invariant kinetic energy, the second contribution corresponds to the mass term, and the last part gives the color electric energy.

For quantum (and classical) simulation purposes, it is more convenient to work with a discretised version of the continuum Hamiltonian, which is defined on a spatial lattice whose points are separated by a distance a_l. In this work, we adopt the staggered formulation of Kogut and Susskind, where fermions and antifermions occupy separate lattice sites, and are arranged in an alternating pattern along the lattice. This yields the lattice Hamiltonian

$$\begin{aligned}\hat{H}_l =& \frac{1}{2a_l}\sum_{n=1}^{N-1}\left(\hat{\phi}_n^\dagger \hat{U}_n \hat{\phi}_{n+1} + \text{H. C.}\right) \\ &+ m\sum_{n=1}^{N}(-1)^n \hat{\phi}_n^\dagger \hat{\phi}_n + \frac{a_l g^2}{2}\sum_{n=1}^{N-1}\hat{\boldsymbol{L}}_n^2.\end{aligned} \tag{2}$$

The matter field at each lattice site n is described by a two-component fermionic field $\hat{\phi}_n = (\hat{\phi}_n^1, \hat{\phi}_n^2)^T$, where the upper index labels the two possible colors. These are related to the components of the continuum spinor in Supplementary Eq. (1) in the limit of vanishing lattice spacing $a_l \to 0$ as $i^p(-1)^n\hat{\phi}_{2n+p}/\sqrt{a_l} \to \hat{\psi}_{p+1}(z)$ with $p = 0, 1$. In the first (kinetic) term, the parallel transporter (or connection) $\hat{U}_n = \exp\left(i\hat{\Omega}_n^a T^a\right)$ acts on the link between sites n and $n+1$ and mediates the interaction between the internal color degree of freedom of the fermions on neighbouring sites. Its presence ensures the invariance of the Hamiltonian under local gauge transformations. The angular variables $\hat{\Omega}_n^a$ are related to the continuum gauge field on the link n as $\hat{\Omega}_n^a/(a_l g) \to \hat{A}_1^a(z)$ when the lattice spacing goes to zero.

The last term in the Hamiltonian corresponds to the invariant Casimir operator of the theory and represents the color electric field energy stored in the gauge links. More precisely, $\hat{\boldsymbol{L}}_n^2 = \hat{L}_n^a\hat{L}_n^a = \hat{R}_n^a\hat{R}_n^a$ where $\hat{L}_n^a$ and $\hat{R}_n^a$ (with $a = x, y, z$) are respectively the left and right color electric field components on link n. They are conjugate

† yyatas@uwaterloo.ca
‡ jingleizl@gmail.com
§ jan.frhaase@gmail.com

momenta of the vector potential [2] and are related to the continuum variable via $\hat{L}_n^a \to \hat{L}^a(z)/g$. The operators $\hat{L}_n^a$ and $\hat{R}_n^a$ satisfy the algebra $[\hat{R}_n^a, \hat{R}_m^b] = i\epsilon_{abc}\hat{R}_n^c\delta_{mn}$, $[\hat{L}_n^a, \hat{L}_m^b] = -i\epsilon_{abc}\hat{L}_n^c\delta_{mn}$, and $[\hat{L}_n^a, \hat{R}_m^b] = 0$, where ϵ_{abc} is the Levi-Civita symbol. For a non-Abelian gauge group, the right and left color electric field are related via the adjoint representation $\hat{R}_n^a = (\hat{U}_n^{\text{adj}})_{ab}\hat{L}_n^b$, with

$$(\hat{U}_n^{\text{adj}})_{ab} = 2\text{Tr}\left[\hat{U}_n T^a \hat{U}_n^\dagger T^b\right]. \tag{3}$$

Concluding, if one follows the mapping between the lattice and continuum variables prescribed above, and employs the correspondence $a_l \sum_{n=1}^N f(a_l n) \to \int \mathrm{d}z\, f(z)$ as $a_l \to 0$, one can recover the continuum Hamiltonian in Supplementary Eq. (1) from the lattice version given in Supplementary Eq. (2).

Supplementary Note 2. Elimination of the gauge fields

For one spatial dimension and open boundary conditions the gauge fields can be eliminated and expressed in terms of the fermionic fields [3, 4]. The decoupling is achieved by a unitary transformation $\hat{\Theta}$ that acts on the fermionic fields and eliminates the gauge connections $\hat{U}_n$ from the kinetic term and expresses the color electric energy in terms of the fermionic operators. This approach was recently introduced by the authors of [4], who performed tensor-network simulations of the SU(2) gauge theory to study real time dynamics of string breaking phenomena. In the following, we reproduce the main steps of their derivation.

We seek a unitary transformation $\hat{\Theta}$ such that $\hat{\Theta}\left(\hat{\phi}_n^\dagger \hat{U}_n \hat{\phi}_{n+1}\right)\hat{\Theta}^\dagger = \hat{\phi}_n^\dagger \hat{\phi}_{n+1}$. Since the $\hat{U}_n$ are unitary, one may demand the action of $\hat{\Theta}$ on the fermionic field to be $\hat{\Theta}\hat{\phi}_n\hat{\Theta}^\dagger = \hat{U}_{n-1}^\dagger \hat{U}_{n-2}^\dagger \cdots \hat{U}_1^\dagger \hat{\phi}_n$. One hence introduces the operators

$$\hat{W}_k = \exp\left(i\,\hat{\boldsymbol{\Omega}}_k \cdot \sum_{m>k} \hat{\boldsymbol{Q}}_m\right), \tag{4}$$

where $\hat{\boldsymbol{Q}}_m$ is the vector of the non-Abelian charges with components

$$\hat{Q}_m^a = \hat{\phi}_m^{i\dagger}(T^a)_{ij}\hat{\phi}_m^j, \quad a = x, y, z. \tag{5}$$

The three (operator) components vector $\hat{\boldsymbol{\Omega}}_k = -a_l g \hat{\boldsymbol{A}}_1$ is directly proportional to the spatial component of the gauge field at site k, and are related to the parallel transporters, since $\hat{U}_k = \exp\left(i\,\hat{\Omega}_k^a T^a\right)$. In [4], it was shown that the desired $\hat{\Theta}$-transformation is explicitly given by $\hat{\Theta} = \hat{W}_1\hat{W}_2\cdots\hat{W}_N$. Under this transformation, the Kogut-Susskind Hamiltonian given by Supplementary Eq. (2) takes the form

$$\hat{H}_{\text{rot}} \equiv \hat{\Theta}\hat{H}\hat{\Theta}^\dagger = \frac{1}{2a_l}\sum_{n=1}^{N-1}\left(\hat{\phi}_n^\dagger\hat{\phi}_{n+1} + \text{H.C.}\right) + m\sum_{n=1}^{N}(-1)^n\hat{\phi}_n^\dagger\hat{\phi}_n + \frac{a_l g^2}{2}\hat{H}_e, \tag{6}$$

where the rotated color electric term is given by

$$\hat{H}_e \equiv \sum_{n=1}^{N-1}\hat{\Theta}\hat{\boldsymbol{L}}_n^2\hat{\Theta}^\dagger = \sum_{n=1}^{N-1}\left(\hat{\boldsymbol{R}}_0 + \sum_{m\leqslant n}\hat{\boldsymbol{Q}}_m\right)^2, \tag{7}$$

and $\hat{\boldsymbol{R}}_0$ can now be interpreted as a background field.

We now give more details on how Gauss's law has been used in order to arrive at Supplementary Eq. (7). In the initial frame (before the $\hat{\Theta}$ transformation), Gauss's law reads

$$\hat{G}_n^a \equiv \hat{L}_n^a - \hat{R}_{n-1}^a - \hat{Q}_n^a, \quad a = x, y, z, \tag{8}$$

where $\hat{L}_n^a$ and $\hat{R}_{n-1}^a$ act on the links emanating from the site n, which itself carries the non-Abelian color charge $\hat{Q}_n^a$. Gauss's law operators $\hat{G}_n^a$ are the generators of the local gauge transformations, hence we have $[\hat{H}, \hat{G}_n^a] = 0, \forall n, \forall a$. Since we assume that there are no external charges in the system, the gauge invariant states must satisfy the identity $\hat{L}_n^a - \hat{R}_{n-1}^a = \hat{Q}_n^a$.

In order to see how Gauss's law transforms under $\hat{\Theta}$, one needs to find the transformation rules for $\hat{L}_n^a$, $\hat{Q}_n^a$, and $\hat{R}_{n-1}^a$. Using the following commutation relation [5]

$$[\hat{L}_n^a, (\hat{U}_n)_{pq}] = (T^a\hat{U}_n)_{pq}, \tag{9}$$

and recognising that $\hat{W}_n$ has the same matrix structure as $\hat{U}_n$, we find [4]

$$[\hat{L}_n^a, \hat{W}_n] = \left(\sum_{m>n}\hat{Q}_m^a\right)\hat{W}_n, \tag{10}$$

which leads to

$$\hat{W}_n\hat{L}_n^a\hat{W}_n^\dagger = \hat{L}_n^a - \sum_{m>n}\hat{Q}_m^a. \tag{11}$$

As a consequence, under the $\hat{\Theta}$ transformation the left electric field transforms as

$$\hat{\Theta}\hat{L}_n^a\hat{\Theta}^\dagger = \hat{L}_n^a - \sum_{m>n}\hat{W}_1\cdots\hat{W}_{n-1}\hat{Q}_m^a\hat{W}_{n-1}^\dagger\cdots\hat{W}_1^\dagger. \tag{12}$$

To find how the non-Abelian charges transform under $\hat{W}_k$, we make use of the following identity

$$\hat{W}_k\hat{\phi}_n^i\hat{W}_k^\dagger = (\hat{U}_k)_{ij}\hat{\phi}_n^j, \tag{13}$$

which can be easily derived from the commutation relation $[\hat{Q}_n^a, \hat{\phi}_m^i] = -\delta_{mn}(T^a)_{ij}\hat{\phi}_n^j$. By using the definition

of the non-Abelian charges in terms of the fermionic field given in Supplementary Eq. (5), we find

$$\hat{W}_k \hat{Q}^a_m \hat{W}^\dagger_k = \hat{\phi}^{\dagger i}_m (\hat{U}_k)_{ip} (T^a)_{pq} (\hat{U}^\dagger_k)_{qj} \hat{\phi}^j_m = (\hat{U}^{\mathrm{adj}}_k)_{ab} \hat{Q}^b_m, \tag{14}$$

where the last equality was obtained from the definition of the adjoint representation given in Supplementary Eq. (3), along with the following identity on the generators of SU(2) [6]

$$(T^a)_{ij}(T^a)_{kl} = \frac{1}{2}\left(\delta_{il}\delta_{jk} - \frac{1}{2}\delta_{ij}\delta_{kl}\right). \tag{15}$$

Finally, combining all the results above we can write the transformation of the left electric field as

$$\hat{\Theta}\hat{L}^a_n\hat{\Theta}^\dagger = \hat{L}^a_n - \sum_{m>n}\left(\hat{U}^{\mathrm{adj}}_{n-1}\hat{U}^{\mathrm{adj}}_{n-2}\cdots\hat{U}^{\mathrm{adj}}_1\right)_{ab}\hat{Q}^b_m. \tag{16}$$

Note that since the first site has no charges to its left, the left electric field on the first site transforms as

$$\hat{\Theta}\hat{L}^a_1\hat{\Theta}^\dagger = \hat{L}^a_1 - \sum_{m>1}\hat{Q}^a_m. \tag{17}$$

The transformation rule of the right electric field can easily be obtained from the relation $\hat{R}^a_n = (\hat{U}^{\mathrm{adj}}_n)_{ab}\hat{L}^b_n$ and Supplementary Eq. (16), one finds

$$\hat{\Theta}\hat{R}^a_n\hat{\Theta}^\dagger = \hat{R}^a_n - \sum_{m>n}\left(\hat{U}^{\mathrm{adj}}_{n}\hat{U}^{\mathrm{adj}}_{n-1}\cdots\hat{U}^{\mathrm{adj}}_1\right)_{ab}\hat{Q}^b_m. \tag{18}$$

From Supplementary Eq. (14), we derive the transformation of the non-Abelian charges as

$$\hat{\Theta}\hat{Q}^a_n\hat{\Theta}^\dagger = \left(\hat{U}^{\mathrm{adj}}_{n-1}\hat{U}^{\mathrm{adj}}_{n-2}\cdots\hat{U}^{\mathrm{adj}}_1\right)_{ab}\hat{Q}^b_n. \tag{19}$$

Gauss's law therefore transforms as

$$\hat{\Theta}\hat{G}^a_n\hat{\Theta}^\dagger = \hat{L}^a_n - \hat{R}^a_{n-1} = 0, \;\forall n > 1. \tag{20}$$

In the rotated frame, Gauss's law can thus be solved recursively and gives

$$\hat{L}^a_n = (\hat{U}^{\mathrm{adj}}_{n-1}\hat{U}^{\mathrm{adj}}_{n-2}\cdots\hat{U}^{\mathrm{adj}}_1)_{ab}\hat{L}^b_1, \tag{21}$$

where we have used the relation between the right and left electric field via the adjoint representation. As previously pointed out, the transformation for the first site of the chain has to be studied on its own. For $n = 1$, Gauss's law transforms as $\hat{\Theta}\hat{L}^a_1\hat{\Theta}^\dagger = \hat{R}^a_0 + \hat{Q}^a_1$, where we have used the fact that the right hand side is invariant under the $\hat{\Theta}$ transformation. In fact, $\hat{R}^a_0$ is invariant, since $\hat{\Theta}$ is independent of $\hat{\Omega}^a_0$ by construction, furthermore $\hat{Q}^a_1$ does not have any charge to its left, and is thus invariant from Supplementary Eq. (19). We now use Supplementary Eq. (17) to write the left electric field on the first site as $\hat{L}^a_1 = \hat{R}^a_0 + \sum_{m\geqslant 1}\hat{Q}^a_m$. Inserting this expression into the integrated version of Gauss's law Supplementary Eq. (21), we obtain

$$\hat{L}^a_n = (\hat{U}^{\mathrm{adj}}_{n-1}\hat{U}^{\mathrm{adj}}_{n-2}\cdots\hat{U}^{\mathrm{adj}}_1)_{ab}\left(\hat{R}^b_0 + \sum_{m\geqslant 1}\hat{Q}^b_m\right), \forall n \geqslant 1, \tag{22}$$

which expresses the left electric field as a product of orthogonal (operator valued) matrices with the total non-Abelian charges. Finally, by inserting this expression on the right hand side of Supplementary Eq. (16) we obtain

$$\hat{\Theta}\hat{L}^a_n\hat{\Theta}^\dagger = (\hat{U}^{\mathrm{adj}}_{n-1}\hat{U}^{\mathrm{adj}}_{n-2}\cdots\hat{U}^{\mathrm{adj}}_1)_{ab}\left(\hat{R}^b_0 + \sum_{m\leqslant n}\hat{Q}^b_m\right), \tag{23}$$

which by virtue of the orthogonal nature of the adjoint representation, leads to the transformed electric field used in Supplementary Eq. (7).

By setting the background field $\hat{\boldsymbol{R}}_0 = 0$, and using the fact that the non-Abelian charges commute on different sites, we can rewrite the chromoelectric energy term appearing in the Hamiltonian as

$$\hat{H}_e = \sum_{n=1}^{N-1}(N-n)\hat{\boldsymbol{Q}}^2_n + 2\sum_{n=1}^{N-2}\hat{\boldsymbol{Q}}_n\cdot\sum_{m=n+1}^{N-1}(N-m)\hat{\boldsymbol{Q}}_m, \tag{24}$$

which exhibits long-range interaction between the non-Abelian charges. Note that although the gauge fields do not appear explicitly, the non-Abelian physics is preserved in this formulation and reflected through the long range exotic interaction between non-Abelian charges. The formula given above is completely general and can be used for both Abelian and non-Abelian models as long as the appropriate expressions for the charges are used. For instance, in the staggered formulation of the Abelian U(1) theory, the electric charge at site n is given by $\hat{Q}_n = \hat{\phi}^\dagger_n\hat{\phi}_n - (1-(-1)^n)/2$. Substituting this expression in Supplementary Eq. (24), we recover the electric energy of the Abelian U(1) Kogut-Susskind Hamiltonian which has been studied both theoretically and numerically [7, 8] and has already been implemented on a quantum computer [9, 10].

In the rotated frame, the Kogut-Susskind Hamiltonian is exclusively represented in terms of fermionic degrees of freedom. Although the gauge fields have been eliminated, their interaction with the matter field has been directly incorporated into the Hamiltonian by virtue of Gauss's law. Furthermore, we emphasize that gauge field observables are still accessible in this approach even though they do not appear explicitly in the transformed Hamiltonian.

Supplementary Note 3. Qubit encoding

In this section, we discuss the transformation from the fermionic Hamiltonian in Supplementary Eq. (2) to a for-

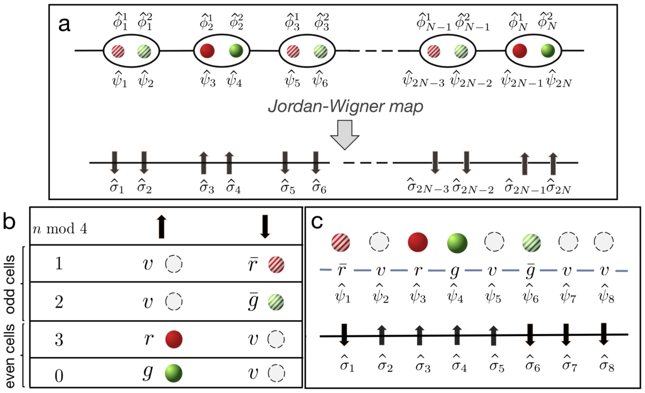

Supplementary Figure 1. **Qubit encoding of the SU(2) Kogut Susskind Hamiltonian. a.** Decolorization of the fermionic field by doubling of the number of lattice sites. Matter (respectively antimatter) sites are represented by filled (respectively stripped) red and green circles. The color degrees of freedom are then encoded in a single component fermionic field $\hat{\psi}_n$. Even sites of the new lattice are occupied by fermions of green color while odd sites host the ones of red color. The single component fermionic fields are subsequently mapped to qubits by performing a Jordan-Wigner transformation. **b.** Translation table between spin and fermion degrees of freedom in the staggered formulation. The matter or antimatter type of a site n is determined by the first column of the table: occupied even cells (defined by $n \bmod 4 = 3, 0$) translate to the presence of matter (red r or green g) while unoccupied odd cells ($n \bmod 4 = 1, 2$) translate to the presence of antimatter (antired $\bar{r}$ or antigreen $\bar{g}$). The vacuum (v) is represented by unoccupied even cells and occupied odd cells. **c.** For illustration, a matter configuration with four spatial spatial sites is shown in the fermion occupation number basis $|\begin{smallmatrix}\bar{r} & r & v & v\\ v & g & \bar{g} & v\end{smallmatrix}\rangle$ (upper panel) and after mapping the fermion fields into Pauli spin operators $|\downarrow\uparrow\uparrow\uparrow\uparrow\downarrow\downarrow\downarrow\rangle$ (lower panel).

mulation that consists of qubits only. The transformation is achieved in two steps: first, the size of the lattice is doubled and the colored fermionic fields are distributed among the new lattice sites by defining the single component fields $\hat{\psi}_{2n-1} = \hat{\phi}^1_n$, $\hat{\psi}_{2n} = \hat{\phi}^2_n$, $n = 1, 2, \ldots, N$. Note that each site now hosts one fermion with a definite color, instead of two fermions of different color. As illustrated in Supplementary Fig. 1a, odd (even) sites of the new lattice are occupied by fermions of red (green) color. By construction, the new field is of fermionic nature as it just corresponds to a relabelling of the existing fermionic fields. The color degree of freedom has thus been absorbed at the cost of doubling the size of the lattice. To have an easier interpretation, the new lattice can be divided in cells $(\hat{\psi}_{2n-1}, \hat{\psi}_{2n})$, with $n = 1, 2, \ldots, N$. For n odd, the fields represent antimatter particles, while cells with even n represent matter particles (see Supplementary Fig. 1b). The parity of the sites thus encodes the color degree of freedom, while the parity of the cells determines the matter-antimatter nature of the particles on the site.

In the second step, the single component fermionic field $\hat{\psi}_n$ is mapped into $\frac{1}{2}$-spin operators by means of a Jordan-Wigner transformation [11]

$$\hat{\psi}_n = \prod_{l<n} (-\hat{\sigma}^z_l)\, \hat{\sigma}^-_n, \quad \hat{\psi}^\dagger_n = \prod_{l<n} (-\hat{\sigma}^z_l)\, \hat{\sigma}^+_n, \tag{25}$$

where $\hat{\sigma}^\pm_n = (\hat{\sigma}^x_n \pm i\hat{\sigma}^y_n)/2$. The string factor $\prod_{l<n}(-\hat{\sigma}^z_l)$ permits to recover the correct fermionic anticommutation relations for the field $\hat{\psi}_n$. Writing the rotated Hamiltonian in Supplementary Eq. (6) in terms of the fermionic field $\hat{\psi}_n$ and applying the Jordan-Wigner transform, we find that the Kogut-Susskind Hamiltonian takes the form

$$\hat{H} = x\tilde{m}\hat{H}_{\mathrm{m}} + \hat{H}_{\mathrm{el}} + x\hat{H}_{\mathrm{kin}}, \tag{26}$$

where we have defined the dimensionless Hamiltonian parameters $\tilde{m} = a_l m$, $x = \frac{1}{a_l^2 g^2}$, and added a constant to normalize the ground state energy to zero in the limit of $x \to 0$. The explicit form of the kinetic term is given by

$$\hat{H}_{\mathrm{kin}} = -\sum_{n=1}^{N-1} \left(\hat{\sigma}^+_{2n-1}\hat{\sigma}^z_{2n}\hat{\sigma}^-_{2n+1} + \hat{\sigma}^+_{2n}\hat{\sigma}^z_{2n+1}\hat{\sigma}^-_{2n+2} + \mathrm{H.C.}\right), \tag{27}$$

the mass term reads

$$\hat{H}_{\mathrm{m}} = 2\sum_{n=1}^{N} \left(\frac{(-1)^n}{2}\left(\hat{\sigma}^z_{2n-1} + \hat{\sigma}^z_{2n}\right) + 1\right), \tag{28}$$

and finally, the chromoelectric Hamiltonian is expressed as

$$\begin{aligned}\hat{H}_{\mathrm{el}} = {} & \frac{3}{8}\sum_{n=1}^{N-1}(N-n)(1-\hat{\sigma}^z_{2n-1}\hat{\sigma}^z_{2n}) \\ & + \frac{1}{8}\sum_{n=1}^{N-2}\sum_{m>n}^{N-1}(N-m)\left(\hat{\sigma}^z_{2n-1} - \hat{\sigma}^z_{2n}\right)\left(\hat{\sigma}^z_{2m-1} - \hat{\sigma}^z_{2m}\right) \\ & + \sum_{n=1}^{N-2}\sum_{m>n}^{N-1}(N-m)\left(\hat{\sigma}^+_{2n-1}\hat{\sigma}^-_{2n}\hat{\sigma}^+_{2m}\hat{\sigma}^-_{2m-1} + \mathrm{H.C.}\right).\end{aligned} \tag{29}$$

The gauge fields do no longer appear explicitly in the new formulation at the expense of introducing long range spin-spin interactions that are present in the chromoelectric Hamiltonian. Crucially, some of these terms are off-diagonal interactions, and are a direct consequence of the non-Abelian nature of the model, and do not appear for instance in the U(1) Abelian Schwinger model. The qubit formulation of the SU(2) Kogut-Susskind Hamiltonian opens the way to the implementation of a non-Abelian model containing both matter and gauge fields on a quantum computer.

Supplementary Note 4. Symmetries and eigenstates

In this section we discuss the structure of the eigenstates of the qubit Hamiltonian given in Supplementary

Eq. (26), considering the symmetry constraints of the theory.

Since we consider the neutral charge sector, we can expand the eigenstates of the Hamiltonian in the basis of the zero mode eigenstates of the total non-Abelian charges, which in the qubit formulation read

$$\hat{Q}^x_{\rm tot} = \frac{1}{2}\sum_{n=1}^{N}\left(\hat{\sigma}^+_{2n-1}\hat{\sigma}^-_{2n} + {\rm H.C}\right), \tag{30}$$

$$\hat{Q}^y_{\rm tot} = \frac{i}{2}\sum_{n=1}^{N}\left(\hat{\sigma}^-_{2n-1}\hat{\sigma}^+_{2n} - {\rm H.C}\right), \tag{31}$$

$$\hat{Q}^z_{\rm tot} = \frac{1}{4}\sum_{n=1}^{N}\left(\hat{\sigma}^z_{2n-1} - \hat{\sigma}^z_{2n}\right), \tag{32}$$

while the baryon number is given by

$$\hat{B} = \frac{1}{4}\sum_{n=1}^{2N}\hat{\sigma}^z_n. \tag{33}$$

The z-component of the total non-Abelian charge is diagonal, therefore it is easy to find the eigenstates with eigenvalue zero. We note that the action of $\hat{Q}^z_{\rm tot}$ on a cell with spins pointing in the same direction gives zero, and the only non-zero contribution comes from cells with antiparallel spins. In particular, $\hat{Q}^z_{\rm tot}\left|\uparrow\downarrow\right\rangle_k = \frac{1}{2}\left|\uparrow\downarrow\right\rangle_k$ and $\hat{Q}^z_{\rm tot}\left|\downarrow\uparrow\right\rangle_k = -\frac{1}{2}\left|\downarrow\uparrow\right\rangle_k$, where $\left|\uparrow\downarrow\right\rangle_k$ corresponds to the spin configuration at the spatial site (or cell index) k. The first qubit in the ket $\left|\uparrow\downarrow\right\rangle_k$ is thus at position $2k-1$ and the second one at position $2k$ of the encoded lattice. As a consequence, in order to be an eigenstate of $\hat{Q}^z_{\rm tot}$ with eigenvalue zero, a basis state must contain as many cells of type $\left|\uparrow\downarrow\right\rangle$ as $\left|\downarrow\uparrow\right\rangle$. If we call $n_{\downarrow\uparrow}$ the number of such cells appearing in the basis state and $n_{\downarrow\downarrow}$ the number of cells with both spins pointing down, then it is easy to see that the baryon quantum number of such state is $B = N/2 - n_{\downarrow\downarrow} - n_{\downarrow\uparrow}$ with $n_{\downarrow\uparrow} = 0, 1, \ldots, N/2$ and $n_{\downarrow\downarrow} = 0, 1, \ldots, N - 2n_{\downarrow\uparrow}$. This clearly shows that the baryon quantum number of a physical state is an integer $B = -N/2, -N/2+1\ldots, N/2$.

Among all the eigenstates of $\hat{Q}^z_{\rm tot}$ with eigenvalue zero, some of them must be combined in order to be annihilated by the two other non-Abelian charges. This is obvious from the expression Supplementary Eq. (30) and Supplementary Eq. (31), where we see that these operators induce non-diagonal transitions between the computational basis states. As a simple illustration, let us consider the state $\left|\uparrow\downarrow\downarrow\uparrow\uparrow\uparrow\uparrow\uparrow\right\rangle$. It contains one cell of type $\left|\uparrow\downarrow\right\rangle$ and one cell $\left|\downarrow\uparrow\right\rangle$ and is thus an eigenstate of $\hat{Q}^z_{\rm tot}$ with eigenvalue zero as per our discussion above. It is however easy to see that this state alone is not an eigenstate of the two other non-Abelian charges $\hat{Q}^{x,y}_{\rm tot}$. In order to be a simultaneous eigenstate of the three non-Abelian charges, it must be combined with its companion state in the table in the following way $\left(\left|\uparrow\downarrow\downarrow\uparrow\uparrow\uparrow\uparrow\uparrow\right\rangle - \left|\downarrow\uparrow\uparrow\downarrow\uparrow\uparrow\uparrow\uparrow\right\rangle\right)/\sqrt{2}$. For an arbitrary number of sites N, the construction discussed above is a non-trivial task. This is why the design of a circuit incorporating the neutral charge constraint is generally harder and one can rely on the variational algorithm to restore the right color symmetry. This approach was for instance used to obtain our results in the $N = 6$ case (see Methods). For small lattice sizes, it is however possible to impose directly the color symmetry into the design of the circuit, as we did in Methods for the circuit generating the color symmetric ansatz for $N = 4$ spatial sites in the sector with baryon number $B = 1$.

[1] Jackiw, R. Introduction to the Yang-Mills quantum theory. *Reviews of Modern Physics* **52**, 661 (1980).

[2] Kogut, J. B. An introduction to lattice gauge theory and spin systems. *Rev. Mod. Phys.* **51**, 659–713 (1979).

[3] Hamer, C. J. Lattice model calculations for SU(2) Yang-Mills theory in 1+1 dimensions. *Nucl. Phys. B* **121**, 159–175 (1977).

[4] Sala, P. *et al.* Variational study of U(1) and SU(2) lattice gauge theories with gaussian states in 1+1 dimensions. *Phys. Rev. D* **98**, 034505 (2018).

[5] Kogut, J. & Susskind, L. Hamiltonian formulation of Wilson's lattice gauge theories. *Phys. Rev. D* **11**, 395–408 (1975).

[6] Haber, H. E. Useful relations among the generators in the defining and adjoint representations of SU(N). *SciPost Phys. Lect. Notes* 21 (2021).

[7] Hamer, C. J., Weihong, Z. & Oitmaa, J. Series expansions for the massive Schwinger model in Hamiltonian lattice theory. *Phys. Rev. D* **56**, 55–67 (1997).

[8] Bañuls, M. C., Cichy, K., Cirac, J. I., Jansen, K. & Kühn, S. Density induced phase transitions in the schwinger model: A study with matrix product states. *Phys. Rev. Lett.* **118**, 071601 (2017).

[9] Muschik, C. *et al.* U(1) Wilson lattice gauge theories in digital quantum simulators. *New J. Phys.* **19**, 103020 (2017).

[10] Martinez, E. A. *et al.* Real-time dynamics of lattice gauge theories with a few-qubit quantum computer. *Nature* **534**, 516–519 (2016).

[11] Jordan, P. & Wigner, E. Über das Paulische Äquivalenzverbot. *Zeitschrift fur Physik* **47**, 631–651 (1928).
